# Experimenting with every American king

**DOI:** 10.1007/s11050-023-09211-2

**Published:** 2023-10-26

**Authors:** Poppy Mankowitz

**Affiliations:** University of Bristol, Bristol, United Kingdom

**Keywords:** Every, Quantifiers, Empty restrictors, Experiment

## Abstract

The standard contemporary semantics for ‘every’ predict the truth of occurrences of sentences with restrictors that denote the empty set, such as ‘Every American king lives in New York’. The literature on empty restrictors has been concerned with explaining a particular violation of this prediction: many assessors consider empty-restrictor sentences to be odd rather than valued, and they are apparently more likely to do so when such sentences include determiners like ‘every’ as opposed to those like ‘no’. Empirical investigation of this issue is overdue, and I present the results of three experimental surveys. The first unexpected outcome is that there is no evidence of a contrast in assessors’ tendencies to judge sentences to be odd based on determiner type. An additional surprising result is that those assessors who assign a truth value to sentences where ‘every’ combines with an empty restrictor overwhelmingly assign the value false. The full results do not fit straightforwardly with any existing account.

## Introduction

*Determiners* (‘every’, ‘no’, ‘the’, ‘most’, ‘some’, ‘three’, etc.) sometimes combine with *empty restrictors* (noun phrase arguments that denote the empty set at the relevant context). Given that America is currently a republic, ‘American king’ is an empty restrictor when (1-a) and (1-b) occur relative to the actual world and time:

1.
  - Every American king lives in New York.
  - No American king lives in New York.^1^

Standard contemporary analyses of determiners predict that occurrences of (1-a) and (1-b) should be unproblematically true. Yet many assessors are reluctant to assign truth values in such cases, instead experiencing a sense of oddness. The literature on empty restrictors has additionally focused on explaining a putative contrast between two types of determiner, whereby assessors are more likely to assign truth values to sentences like (1-b) than to (1-a). The current paper undertakes an empirical investigation of these types of sentences.

According to standard contemporary analyses of determiners, the emptiness of a restrictor does not prevent an occurrence of a sentence from having a binary truth value.^2^ For instance, Generalized Quantifier Theory (Mostowski 1957, Lindström 1966, Montague 1973, Barwise and Cooper 1981) predicts that whenever ‘no’ combines with an empty restrictor, the occurrence of the sentence will be true, since the intersection of the empty set and the second argument of the quantifier will be empty. It also predicts that whenever ‘every’ combines with an empty restrictor, the occurrence of the sentence will be true, since the empty set will be a subset of the second argument of the quantifier. This is clear from the semantics assigned to ‘every’ by Generalized Quantifier Theory and other standard contemporary approaches:

- **Standard contemporary semantics:** 〚Every NP VP〛 = 1 iff 〚NP〛 ⊆ 〚VP〛.

An extensive literature on empty restrictors (Horn 1977, de Jong and Verkuyl 1984, Lappin and Reinhart 1988, Moravcsik 1991, Abusch and Rooth 2003, von Fintel 2004, Reinhart 2004, Geurts 2007) has endeavoured to explain the fact that assessors often consider empty-restrictor sentences with ‘every’ to be odd rather than true. Lappin and Reinhart (1988) were the first to make the further claim that assessors’ responses to empty-restrictor sentences vary based on the determiner. They held that, while most assessors find occurrences of sentences with empty restrictors odd when the determiner is *strong* (e.g., ‘every’, ‘most’, ‘the’, ‘the *n*’), many (though not all) assessors assign the predicted truth values when the determiner is *weak* (e.g., ‘no’, ‘some’, ‘many’, ‘*n*’).^3^ In subsequent years, various explanations of this observation have been proposed.

One view is that the semantics of strong determiners should be altered to include a presupposition that the restrictor is non-empty, as a definedness condition (de Jong and Verkuyl 1984; Diesing 1992). Then, the oddness of occurrences of sentences where strong determiners combine with empty restrictors arises because they lack a binary truth value:^4^

- **Non-standard contemporary semantics:** 〚Every NP VP〛 = 1 if 〚NP〛 ⊆ 〚VP〛 & 〚NP〛 ≠∅; = 0 if 〚NP〛 ⊈ 〚VP〛 & 〚NP〛 ≠∅; otherwise undefined.

A more popular strategy is to supplement the standard semantics with the following thesis:

- **Pragmatic oddness:** When 〚NP〛 =∅, an occurrence of ‘Det NP VP’ will have the value assigned by the correct semantics, but might be considered odd by assessors due to some pragmatically infelicitous feature.

The pragmatic feature is claimed to be present for all occurrences of empty-restrictor sentences with strong determiners and for some such occurrences with weak determiners. This pragmatic feature has been identified as a particular processing strategy (Lappin and Reinhart 1988), a non-emptiness presupposition separate from the semantics (Reinhart 2004; Geurts 2007), or an implausible conversational implicature (Abusch and Rooth 2003; Peters and Westerståhl 2006, pp. 125-126). While there is ongoing debate about the plausibility of these proposals, my current concern centres on the data that they attempt to explain.

In a pilot for an experiment investigating this issue, it came to my attention that those assessors who assigned a binary truth value to occurrences of empty-restrictor sentences with ‘every’ near uniformly assigned the value *false*. This was surprising, since judgements of falsity are predicted by neither the standard semantics, nor the non-standard semantics, nor accounts of pragmatic features that cause a sense of oddness. It is well-known that Aristotle and many medieval logicians treated sentences where ‘every’ combines with empty restrictors as false, by virtue of endorsing the following semantics:

- **Aristotelian semantics:** 〚Every NP VP〛 = 1 iff 〚NP〛 ⊆ 〚VP〛 & 〚NP〛 ≠∅.

If assessors have a robust tendency to judge empty-restrictor sentences with ‘every’ to be false, then this might provide some motivation to reconsider the Aristotelian semantics.^5^

I carried out three experiments that were designed to investigate the putative contrast based on determiner strength, and to evaluate the predictions of the three semantic accounts for ‘every’. First, the results provide no evidence for the contrast. Second, while there is evidence that assessors often consider empty-restrictor sentences with ‘every’ to be odd, there is also evidence of a strong tendency to judge them to be false. The full results are difficult to reconcile with any of the semantic accounts, although I evaluate some options for reconciliation.

## The current experiments

### General background

The three accounts of empty-restrictor sentences with ‘every’ are summarised in Table 1. On the face of it, standard semantic accounts predict that assessors will react to such sentences with judgements of truth or oddness responses. The non-standard semantics predict oddness responses. Aristotelian semantic accounts predict judgements of falsity or oddness responses. While I later consider supplements that might help the accounts to accommodate the full results (see §6), it is these predictions that will inform the experimental design and initial discussion of results.

**Table 1:** Analyses of ‘Every NP VP’ with empty ‘NP’

*Semantics*	*Predicted value*	*Supplements for oddness*
*Standard*	true	pragmatic oddness
*Non-standard*	undefined	-
*Aristotelian*	false	pragmatic oddness

To my knowledge, there has been no experimental testing of the relation between assessors’ inclinations to assign truth values and determiner strength. This is despite an increasing emphasis on the application of empirical methods, both in linguistics in general (see Gibson and Fedorenko 2013) and to the processing of determiners in particular (e.g., Villalta 2003; Chemla 2009; Hackl 2009; Ionin 2010; Lidz et al. 2011; Kotek et al. 2015; Geurts and van Tiel 2016; van Tiel et al. 2018; Syrett and Brasoveanu 2019). Lappin and Reinhart’s (1988) observation of a contrast is often simply taken for granted. While some cite their own “cursory field research” (von Fintel 2004) or “informal checking [...] in various classes” (Reinhart 2004) regarding this contrast, the full results of these surveys have not to the best of my knowledge been presented or formally analysed.

There has been some recent experimental research on assessors’ responses to determiners with arguments that denote the empty set. Abrusán and Szendrői (2013) and Schwarz (2016) investigated empty-restrictor sentences with ‘the’, and the former found a high proportion of choices of ‘False’ for some such sentences. Bott et al. (2019) reported evidence of increased processing difficulties for German sentences where translations of determiners like ‘fewer than *n*’ and ‘at most *n*’ combine with empty nuclear scopes, compared to variants with non-empty nuclear scopes. However, none of these prior experiments have tested empty-restrictor sentences with ‘every’ or sought a contrast between strong and weak determiners.

### General methodology

The current experiments consist of surveys where participants were presented with sentences and asked to select ‘True’, ‘False’ or ‘Can’t say’. These labels were intended to correlate with assessors’ psychological responses: respectively, judgements of truth, judgements of falsity, and oddness responses (i.e., disinclinations to assign a binary truth value). Certain methodological choices require further discussion.

Firstly, while some have argued that sentence judgements should only be sought relative to verbal or visual presentations of the envisaged context (e.g., Matthewson 2004; Tonhauser and Matthewson 2016), my surveys followed others (e.g., Abrusán and Szendrői 2013) in presenting isolated sentences. Specification of additional features of the context was avoided for several reasons. First, the literature on empty restrictors does not link oddness responses to any particular type of context: they are supposed to arise relative to whatever strategies speakers use to assess empty-restrictor sentences presented out of context. This motivates testing for oddness responses by allowing participants to deploy the ordinary strategies. Second, while I intended participants to assess the sentences relative to imaginary contexts of utterance at the actual time and world, it seems likely that participants do this despite an isolated presentation of sentences. There is evidence from psycholinguistics that individuals rapidly accommodate a context as they read an isolated sentence, and it is reasonable to expect that the accommodated context will generally reflect features of their current surroundings.^6^ Indeed, all participants whose results were admitted attained a high degree of accuracy for ‘catch’ filler items, and accuracy for such items required assessment relative to the actual time and world. Third, if there is something about empty-restrictor sentences that overrides the general tendency to accommodate contexts at the actual time and world—say, if assessors are likely to imagine an occurrence of the sentence relative to a non-actual context where the restrictor is non-empty (see §6)—then it seems appropriate to allow this process to occur when testing for oddness responses. In sum, testing the predictions of the literature motivates allowing participants to apply their usual assessment strategies to isolated empty-restrictor sentences; and while these strategies might be expected to involve evaluating occurrences relative to contexts at the actual world and time, a strategy of evaluation relative to non-actual worlds should not be blocked if such a strategy is common. On the other hand, future experiments that present empty-restrictor sentences alongside manipulable contexts will be important in building a full picture of the assessment of such sentences (see §6.4).

A second notable methodological choice concerns the lexical differences between conditions. In all of the experiments, there are no instances where the same expression serves as an empty restrictor in one condition and as a non-empty restrictor in another condition, or as a nuclear scope in one condition with an empty restrictor and in another condition with a non-empty restrictor. For instance, in Experiment 1 the restrictor ‘American king’ and the nuclear scope ‘lives in New York’ only occur in the empty-restrictor conditions. This was largely an effect of testing isolated sentences that are closely related to those discussed in the literature (see above). Since contexts could not be used to manipulate expressions’ extensions, the expressions that were viable in one condition typically were not viable in other conditions. However, a limitation of this choice is that the reported effects could potentially be influenced by differences in lexical material that are independent of the manipulated factors. For this reason, we cannot be confident that the reported results will generalise beyond the particular type of items included in the experiments. This limitation could again be addressed by future experiments that present empty-restrictor sentences alongside manipulable contexts (see §6.4).

A final methodological choice of note is the use of the phrase ‘Can’t say’ as the third option. I hoped that participants would choose ‘Can’t say’ if and only if they experienced a sense of oddness that makes them disinclined to assign a truth value. The exact wording was inspired by Abrusán and Szendrői (2013). They selected this label on the basis of pilot studies in which they found no difference between ‘Can’t say’ and alternative options like ‘Neither true nor false’. They were also concerned that ‘Neither true nor false’ would be overly theoretical for an experiment that aims to access assessors’ pre-theoretic linguistic intuitions. That is, it might force participants to consider whether their oddness response indicates a lack of truth value, which might require metasemantic theorising. Another concern about the label ‘Neither true nor false’ is that it is not theory-neutral regarding the source of oddness responses: only the non-standard contemporary semantics take occurrences of empty-restrictor sentences that elicit oddness responses to lack truth values. For these reasons, I selected ‘Can’t say’ as the third option.

One drawback of this approach is that ‘Can’t say’ might also be an appropriate choice if an assessor does not consider a sentence to be odd but lacks the knowledge required to decide whether it is true or false. Given that I deliberately chose targets that relied on very basic general knowledge—that there are no American kings, that all living people were born on Earth, and so on—it seems unlikely that assessors who evaluate the sentences relative to contexts at the actual world would experience epistemic uncertainty. The instructions included an example sentence—‘The square triangle has four sides’—for which I instructed participants to select ‘Can’t say’. I expected this sentence to be considered odd in a way that makes assessors disinclined to assign a truth value, whether or not the cause of the oddness response is the same as for empty-restrictor sentences with ‘every’ and ‘no’. My main aim was to signal that ‘Can’t say’ was a legitimate option for the sentences that participants would encounter, rather than something to be avoided by selecting a truth value in a possibly arbitrary manner. Yet an additional effect is that assessors should have linked a sense of oddness with the selection of ‘Can’t say’. Still, the possibility cannot be excluded that participants linked the selection of ‘Can’t say’ for the example sentence with epistemic uncertainty, and subsequently made this selection predominantly when experiencing epistemic uncertainty rather than oddness. For instance, perhaps many participants were simply unsure whether a ‘square triangle’ was a particular type of mathematical shape that they had never learned about. To ensure that epistemic uncertainty is not leading to choices of ‘Can’t say’, future experiments could pair each target sentence of the form ‘Every A B’ with a control sentence of the form ‘There are some As’ (e.g., ‘There are some American kings’), with participants who fail to select ‘False’ for these controls’ being excluded from the analysis. Moreover, if it turned out that a significant majority of participants passed this inclusion criterion, then this might provide some evidence that participants in the current experiments were unlikely to have selected ‘Can’t say’ due to epistemic uncertainty.

## Experiment 1

### Methods

#### Participants

Fifty-four English speakers living in the United States were recruited through Amazon Mechanical Turk. Each gave informed consent and received a small fee upon satisfactory completion of an online survey.^7^

#### Materials

I designed a 2 × 3 factorial experiment. The first factor for which the target sentences varied was the inclusion of the strong determiner ‘every’ (∀) and the weak determiner ‘no’ (¬∃). A single determiner of each type was used in order to limit processing demands. The second factor was the inclusion of an empty restrictor ($R_{ \emptyset}$), a non-empty restrictor that combines with ‘every’ to produce sentences that the standard semantics predict to be true (*T*; note that these sentences are predicted to be false when the determiner is ‘no’), and a non-empty restrictor that combines with ‘every’ to produce sentences that the standard semantics predict to be false (*F*; these sentences are predicted to be true when the determiner is ‘no’). Each of the six test conditions included eight items. The first item in each condition is given in Table 2, along with the truth value predicted by each of the semantics (see the Appendix for the full table of items and fillers). Note that some versions of the non-standard contemporary semantics predict that ‘no’ combines with empty restrictors to yield truth, whereas other versions predict that ‘no’ has one disambiguation that yields truth and another disambiguation that yields undefinedness (see fn. 4). Each item set consisted of two items that vary with respect to their determiner.

**Table 2:** First item for each condition

*Condition*	*Item*	*Sentence*	*Standard*	*Non-standard*	*Aristotelian*
∀-$R_{\emptyset}$	1	Every American king lives in New York	true	undefined	false
¬∃-$R_{\emptyset}$	1	No American king lives in New York	true	true/undefined	true
∀-*T*	9	Every living person was born on Earth	true	true	true
¬∃-*T*	9	No living person was born on Earth	false	false	false
∀-*F*	17	Every American president was born in China	false	false	false
¬∃-*F*	17	No American president was born in China	true	true	true

I used the *turktools* package (Erlewine and Kotek 2016) to produce Latin Square counterbalanced randomised lists of items. Fifty-four lists, each containing exactly one item from each item set, were derived in this way. This avoided exposing the same participant to a pair of sentences that differed only with respect to the determiner, given the possibility that encountering one version of the sentence would affect the participant’s response to the second version. Hence each of the forty-eight target items appeared on twenty-seven lists. Each list presented twenty-four target items (four in each condition) in a randomised order alongside twelve fillers. The *turktools* package was used to generate surveys from these lists that were uploaded to Amazon Mechanical Turk.

#### Procedure

Participants were instructed to read each sentence and make a selection from the choices ‘False’, ‘Can’t say’ and ‘True’ (see the Appendix for the full instructions).

#### Predictions

Experiment 1 was designed to test two predictions that emerge from the literature on empty restrictors, and a third prediction derived from my own pilot study:

- **Odd Empty Effect:** Choices of ‘Can’t say’ are more likely for $R_{\emptyset}$ conditions than for *T* conditions.
- **Strength Contrast Effect:** Choices of ‘Can’t say’ are more likely for condition ∀-$R_{\emptyset}$ than for condition ¬∃-$R_{\emptyset}$.
- **False ‘Every’ Effect:** Choices of ‘False’ are more likely for condition ∀-$R_{\emptyset}$ than for condition ∀-*T*.

The second prediction follows straightforwardly from the claim that assessors are more likely to consider empty-restrictor sentences to be odd with ‘every’ than with ‘no’. The first prediction is a weak formulation of the claim that assessors tend to consider empty-restrictor sentences to be odd, measured via comparison with an arbitrary category of non-empty-restrictor sentences. The claim could also be interpreted as predicting more choices of ‘Can’t say’ than other choices for $R_{\emptyset}$ conditions. I tested the weaker formulation in order to be maximally charitable to the literature on empty restrictors. The third prediction is a weak formulation of the claim that assessors who assign values to empty-restrictor sentences with ‘every’ typically assign the value false, measured via comparison with true sentences where ‘every’ combines with non-empty restrictors. The claim might also be interpreted as predicting that there will be more choices of ‘False’ than ‘True’ for condition ∀-$R_{\emptyset}$. I again tested the weaker formulation, in the pursuit of parity.

### Results

**Fig. 1:**
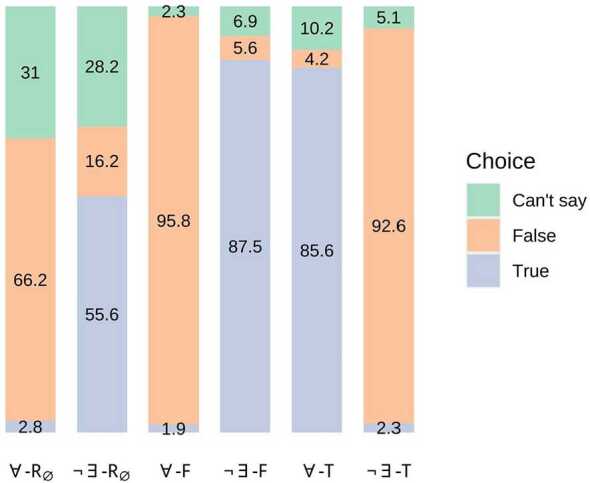
Experiment 1 Results (proportion (%) of choice for each condition; multiplying by 216 and rounding to the nearest integer reconstructs exact counts)

The results (see Fig. 1) show that the proportion of choices of ‘Can’t say’ was higher for the $R_{\emptyset}$ conditions (around 30%) than for the other conditions (between 2.3% and 10.2%), as predicted by the literature on empty restrictors. Moreover, the majority of choices for empty-restrictor sentences with ‘every’ were ‘Can’t say’ (31%) or ‘False’ (66.2%).

I fit mixed-effect multinomial baseline logit models using the *mclogit* package (Elff 2021) with marginal quasi-likelihood approximation, in the software package *R* (R Core Team 2018). These types of models are useful when one wants to analyse a dependent variable that is categorical with more than two levels—such as ‘False’, ‘Can’t say’ and ‘True’—whilst taking account of the variance introduced by participants and items. Multinomial baseline logit models give values for each level of the dependent variable relative to an arbitrary baseline level, with respect to each category of the fixed and random effects; in this case, the models calculate the likelihood of choices of ‘Can’t say’ relative to ‘False’ and the likelihood of ‘True’ relative to ‘False’. I started by fitting a model with the minimal random effects structure justified by the experimental design—random intercepts for participants and items—and used likelihood ratio tests to check whether the addition of random slopes by participant or by item for any of the independent variables improved the fit of the model. In each case, either the model did not converge or there was no significant improvement of fit, so no further random effects were added. Determiner type, restrictor type and their interaction were added as fixed effects. Likelihood ratio tests indicated that the addition of each fixed effect produced a significantly better fit, relative to models without that effect (in each case, *p*<0.0001). Within the full model, the estimated coefficients (given in log-odds) for all but one level of the factors and their interaction attained significance (see Tables 3 and 4). The exception was the difference between restrictor types $R_{\emptyset}$ and *F* for determiner type ¬∃ in the comparison of choices ‘Can’t say’ and ‘False’ (*p* = 0.6052). Hence the model indicates the significance of determiner type, restrictor type and their interaction as predictors for assessors’ choices, even accounting for variance between participants and items.

**Table 3:** ‘Can’t say’ vs. ‘False’ in the model for Experiment 1

	*β*	*SE*	*z value*	*p value*
(Intercept)	0.5555	0.4193	1.325	
∀	−1.3137	0.5342	−2.459	0.0139
*T*	−3.4559	0.5995	−5.765	<0.0001
*F*	−0.3324	0.6430	−0.517	0.6052
∀:*T*	5.1079	0.8697	5.873	<0.0001
∀:*F*	−2.6327	0.9267	−2.841	0.0045

**Table 4:** ‘True’ vs. ‘False’ in the model for Experiment 1

	*β*	*SE*	*z value*	*p value*
(Intercept)	1.2321	0.3504	3.517	
∀	−4.4032	0.5894	−7.471	<0.0001
*T*	−4.9210	0.6154	−7.997	<0.0001
*F*	1.5247	0.5122	2.977	0.002911
∀:*T*	11.1152	0.8976	12.384	<0.0001
∀:*F*	−2.3000	0.9097	−2.528	0.011461

To evaluate the predictions, pairwise comparisons of estimated marginal means were calculated from the full model, using the *emmeans* package (Lenth 2021) with Tukey adjustment. While multinomial baseline logit models compare each response to a baseline response category (in this case, ‘Can’t say’ versus ‘False’ and ‘True’ versus ‘False’), these pairwise comparisons allow each response to be compared to another (e.g., ‘Can’t say’ versus ‘True’) or compared to all of the others (e.g., ‘Can’t say’ versus ‘True’ or ‘False’). This is helpful when evaluating specific hypotheses that go beyond the mere significance of the fixed effects; for instance, the Odd Empty and Strength Contrast predictions concern the likelihood of ‘Can’t say’ relative to any other choice across conditions, not just the likelihood of ‘Can’t say’ relative to the arbitrary baseline level. Mean differences (*MD*) are given on the probability scale, and a positive value indicates an increased likelihood of the relevant choice for one condition compared with the other (see Tables 18 and 19 in the Appendix). Choices of ‘Can’t say’ were significantly more likely for $R_{\emptyset}$ conditions than for *T* conditions (*MD* = 0.22, *z* = 3.32, *p* = 0.0026). The results therefore provide evidence of an Odd Empty Effect. There was no significant difference between the likelihood of choices of ‘Can’t say’ for conditions ∀-$R_{ \emptyset}$ and ¬∃-$R_{\emptyset}$ (*MD* = 0.03, *z* = 0.24, *p* = 0.9999). Hence the differing proportions of choices of ‘Can’t say’ for empty-restrictor sentences with ‘every’ (31.01%) versus ‘no’ (28.24%) was insufficient to support a Strength Contrast Effect. Choices of ‘False’ were significantly more likely for condition ∀-$R_{\emptyset}$ than for ∀-*T* (*MD* = 0.62, *z* = 7.54, *p*<0.0001), which supports a False ‘Every’ Effect.

### Discussion

Experiment 1 provides evidence for the Odd Empty Effect that has been discussed in the literature. However, a stronger prediction—that there will be significantly more choices of ‘Can’t say’ than other choices for conditions ∀-$R_{\emptyset}$ and ¬∃-$R_{\emptyset}$ (see §3.1.4)—is incompatible with the results. The fact that a weak formulation of the claim is supported by the data, but a strong formulation is not, indicates that the literature may have overstated assessors’ propensities to find empty-restrictor sentences odd. Experiment 1 does not provide evidence of a Strength Contrast Effect, which calls into question the view that the likelihood of oddness responses depends on determiner strength. Experiment 1 also provides evidence for a False ‘Every’ Effect. In this case, even the stronger prediction—that there will be more choices of ‘False’ than ‘True’ for condition ∀-$R_{\emptyset}$—attains support. In fact, a comparison of the estimated marginal means for each choice within condition ∀-$R_{\emptyset}$ indicates a significantly greater likelihood of choices of ‘False’ than ‘True’ (*MD* = 0.63, *z* = 7.87, *p*<0.0001), and of choices of ‘Can’t say’ than ‘True’ (*MD* = 0.28, *z* = 3.19, *p* = 0.0041; see Table 22 in the Appendix). The negligible proportion of choices of ‘True’ for empty-restrictor sentences with ‘every’ is surprising, given the predictions of the standard contemporary semantics.

At this point, it is worth considering whether the fact that a notable proportion of assessors chose to select ‘False’ rather than ‘Can’t say’ for empty-restrictor sentences with ‘every’ requires explanation. An alternative hypothesis would be the following:

- **Indistinct choices:** Assessors do not reliably distinguish between choices of ‘Can’t say’ and ‘False’, at least for empty-restrictor sentences.

The hypothesis suggests that assessors’ choices of ‘False’ and ‘Can’t say’ in the empty-restrictor conditions fail to correlate with different types of psychological response in a systematic way. This hypothesis would lead us to expect similar proportions of choices of ‘False’, relative to the combined total of choices of ‘Can’t say’ and ‘False’, for conditions ∀-$R_{\emptyset}$ and ¬∃-$R_{\emptyset}$. However, the results falsify this prediction. This becomes clear simply from examining the model’s coefficients for the comparison of choices of ‘Can’t say’ to the baseline response category ‘False’, since ¬∃ and $R_{\emptyset}$ were used as the model’s reference determiner and restrictor type (the levels relative to which other levels are compared). Choices of ‘Can’t say’ were significantly *less* likely compared with choices of ‘False’ for condition ∀-$R_{\emptyset}$ than for ¬∃-$R_{\emptyset}$ (*β* = −1.31, *z* = −2.46, *p* = 0.0139). This suggests that assessors are making a systematic choice when they select ‘False’ or ‘Can’t say’ for empty-restrictor sentences.

A further defence of the indistinct choices hypothesis might begin with the observation that the proportion of choices of ‘False’ for ¬∃-$R_{\emptyset}$ (16.20%) is higher than the proportion of choices of ‘False’ for sentences where ‘no’ combines with non-empty restrictors that the semantics predict to be true, namely those in condition ¬∃-*F* (5.56%). This difference could be attributed to the greater likelihood of oddness responses in the former case: perhaps a notable proportion of participants selected ‘False’ for empty-restrictor sentences with ‘no’ because they considered them to be odd. This tendency might then be expected to hold for empty-restrictor sentences in general. However, this further defence of the indistinct choices hypothesis does not succeed. First, the estimated marginal means indicate that the difference between the likelihood of choices of ‘False’ for conditions ¬∃-$R_{\emptyset}$ and ¬∃-*F* was not statistically significant (*MD* = 0.11, *z* = 2.48, *p* = 0.1302). A further crucial point is the following: even if the results for condition ¬∃-$R_{\emptyset}$ were to provide evidence that *some* choices of ‘False’ are attributable to oddness responses, it is hard to see how they could justify attributing *all* or even *most* choices of ‘False’ for condition ∀-$R_{ \emptyset}$ to oddness responses.^8^ The results thus indicate that there is something systematic and significant about assessors’ selection of ‘False’ for empty-restrictor sentences with ‘every’. All accounts must explain this.

The False ‘Every’ Effect cannot be explained by the versions of the standard or non-standard contemporary semantics under current consideration (see Table 1). Of course, drawing on further explanatory resources might produce accounts based on the contemporary semantics that are compatible with the results of Experiment 1 (see §6). Yet the fact that the results conform with the predictions of the Aristotelian account—the False ‘Every’ Effect is explained by the semantics; the Odd Empty Effect is explained by the pragmatic oddness thesis—motivates further investigation of these semantics.

## Experiment 2

### Background

Advocates of the standard contemporary semantics often concede that the Aristotelian semantics conform with the judgements of some naive assessors. To defend the standard semantics despite this, it is common to observe that no analysis can preserve the totality of our pre-theoretic intuitions for empty-restrictor sentences (e.g., Strawson 1952, pp. 163-170; Peters and Westerståhl 2006, p. 124). Such intuitions supposedly include the views that ‘Every A is B’ is false if there are no As, that ‘Not every A is B’ is true only if there is some A that fails to be B, and that occurrences of the former sentence are false if and only if occurrences of the latter are true.^9^ It is then common to claim that ‘*logical coherence* speaks in favor of the modern interpretation’ (Peters and Westerståhl 2006, p. 27), insofar as important logical properties of quantifiers are preserved by abandoning the first intuition and upholding the second two.

In other words, some advocates of the standard contemporary semantics have argued that accepting the view that (1-a) is true—while using supplements to explain the Odd Empty and False ‘Every’ Effects—is no more counterintuitive than endorsing the Aristotelian semantics, since one would then commit oneself to the position that (2-a) is false and (2-b)–(2-c) are true:

- Every American king lives in New York.
-
  - Every American king is an American king.
  - Not every American king lives in New York.
  - Not every American king is an American king.

The predictions of the non-standard contemporary semantics are more complex: sentences with ‘not every’ will presumably have one reading where the non-emptiness presupposition encoded in the semantics of ‘every’ projects (yielding undefinedness when the restrictor is empty), along with a presupposition-cancelling reading involving ‘external’ negation (yielding truth whenever occurrences of the unnegated sentence fail to be true; see Horn 1985). Still, similar claims of counterintuitive predictions apply. For instance, Lappin and Reinhart (1988, p. 1026) hold that any position denying that (2-a) is true and that (2-c) is false ‘would be very difficult to support on the basis of linguistic intuitions’. Peters and Westerståhl (2006, p. 124) state that the truth of empty-restrictor sentences with ‘not every’ is ‘highly counterintuitive to naive speakers today’.

Experiment 2 aims to evaluate the argument that, once we look at a broader range of empty-restrictor sentences, the intuitive judgements conform better with the values predicted by the standard semantics than the alternatives. One complication is that sentences of the form ‘Not every A is B’ carry a scalar implicature that some A is B (Levinson 1983, p. 134). The fact that this implicature will be false when ‘A’ is an empty restrictor might make assessors reluctant to describe occurrences as ‘true’, even if the occurrences were to be semantically true. Indeed, empirical evidence indicates that a significant proportion of participants in truth value judgement tasks adopt this response strategy for targets with false implicatures (see Spychalska et al. 2016). This makes it difficult to test the predictions of the Aristotelian and non-standard contemporary semantics, at least when participants are not trained to disentangle expressed and implicated content.^10^ Still, if an experiment were to reveal any non-negligible proportion of judgements of truth for such sentences, then this would undermine the argument that the standard semantics clearly capture speakers’ intuitions about ‘not every’.

### Methods

#### Participants

Forty-eight English speakers were recruited through Amazon Mechanical Turk in the same way as for Experiment 1.

#### Materials

I designed a 2 × 2 factorial experiment where the first factor for which the target sentences varied was the inclusion of ‘every’ (∀) and ‘not every’ (¬∀). The second factor was the inclusion of a non-empty *nuclear scope*—the material that follows the restrictor—($R_{\emptyset}$), or an empty nuclear scope ($R_{ \emptyset}=S$). Condition ∀-$R_{\emptyset}$ consisted of the eight sentences from Experiment 1’s condition ∀-$R_{ \emptyset}$, and condition ¬∀-$R_{\emptyset}$ consisted of the sentences from ∀-$R_{\emptyset}$ with the addition of ‘not’. Each sentence in the $R_{\emptyset}=S$ conditions was derived by converting the empty restrictor ‘A’ from a sentence in the $R_{\emptyset}$ conditions into a nuclear scope of the form ‘is an A’. This resulted in four test conditions, with eight items in each test condition. The first item in each condition is given in Table 5, along with the value predicted by the various semantics (see the Appendix for the full list of items and fillers). Each item set consisted of one item with ‘every’ and one with ‘not every’.

**Table 5:** First item for each condition

*Condition*	*Item*	*Sentence*	*Standard*	*Non-standard*	*Aristotelian*
∀-$R_{\emptyset}$	1	Every American king lives in New York	true	undefined	false
¬∀-$R_{\emptyset}$	1	Not every American king lives in New York	false	undefined/true	true
∀-$R_{\emptyset}=S$	9	Every American king is an American king	true	undefined	false
¬∀-$R_{\emptyset}=S$	9	Not every American king is an American king	false	undefined/true	true

Forty-eight lists were derived in the same manner as for Experiment 1, each containing one item from each item set. This avoided exposing the same participant to a pair of sentences that differed only with respect to the first expression. However, I chose to expose each participant to a pair of sentences where the restrictor of the first occurred in both the restrictor and the nuclear scope of the second. That is, each participant received one item from item set 1 with either ‘every’ or ‘not every’ (see Table 5) along with one from item set 9 with the opposite phrase. The aim was to ensure that the emptiness of the restrictors was salient, in order to discourage participants from judging sentences in the $R_{\emptyset}=S$ conditions without contemplating the status of the restrictor. Hence each of the thirty-two target items appeared on exactly twenty-four lists. For each list, sixteen target items (four in each condition) were presented in a randomised order alongside sixteen filler sentences. Surveys were generated from the lists in the same way as for Experiment 1.

#### Procedure

The instructions and presentation of surveys were the same as for Experiment 1.

#### Predictions

Experiment 2 was designed to test the predictions of the various semantics:

- **Standard contemporary predictions:**
  1. Choices of ‘True’ are more likely for ∀-$R_{\emptyset}$ than ¬∀-$R_{\emptyset}$.
  2. Choices of ‘True’ are more likely for ∀-$R_{\emptyset}=S$ than ¬∀-$R_{\emptyset}=S$.
- **Non-standard contemporary and Aristotelian predictions:**
  1. Choices of ‘True’ are more likely for ¬∀-$R_{\emptyset}$ than ∀-$R_{\emptyset}$.
  2. Choices of ‘True’ are more likely for ¬∀-$R_{\emptyset}=S$ than ∀-$R_{\emptyset}=S$.

The predictions of the standard and Aristotelian semantics follow straightforwardly from their expected truth values. The non-standard contemporary semantics issue the same predictions as the Aristotelian semantics because they expect a presupposition-cancelling reading to be available for items with ‘not every’ that will yield the value true. I focused on choices of ‘True’ across pairs of conditions because all accounts that are compatible with Experiment 1’s results—due to endorsing pragmatic supplements that allow them to explain both the False ‘Every’ and Odd Empty Effects—expect there to be some non-negligible proportion of choices of ‘False’ and ‘Can’t say’ across all conditions. Since none of the accounts predict the exact proportion of these choices, it would be difficult to extract predictions about the proportion of choices within each condition, or the proportion of choices of ‘Can’t say’ and ‘False’ across conditions.

### Results

**Fig. 2:**
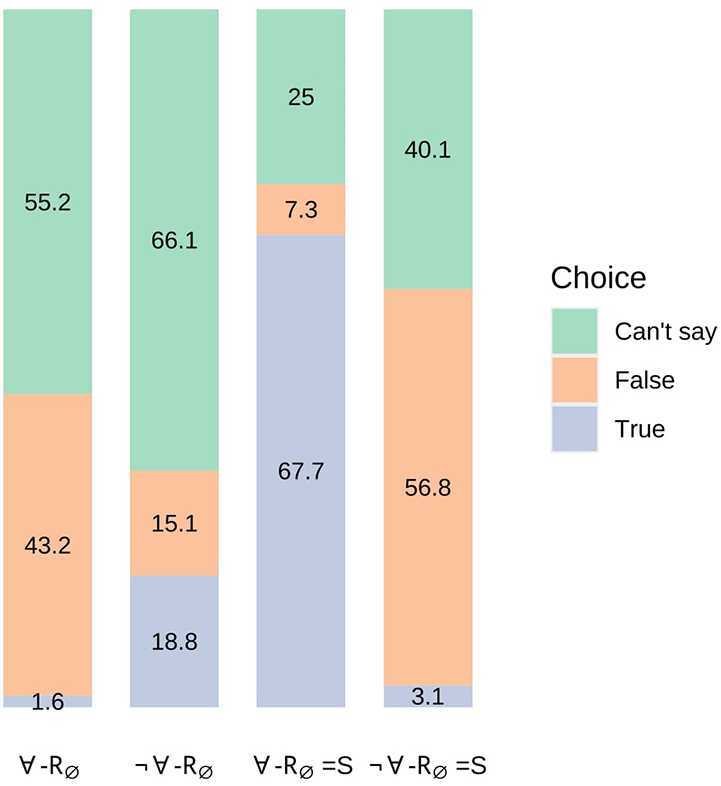
Experiment 2 Results (proportion (%) of choice for each condition; multiplying by 192 and rounding to the nearest integer reconstructs exact counts)

The full results are presented in Fig. 2. As for Experiment 1, the majority of choices for empty-restrictor sentences with non-empty nuclear scopes and ‘every’ were ‘Can’t say’ (55.2%) or ‘False’ (43.2%).^11^ The first prediction of the standard semantics is incompatible with the results: it is not the case that choices of ‘True’ were more likely for ∀-$R_{\emptyset}$ than ¬∀-$R_{\emptyset}$. The second prediction of the alternative semantics is also incompatible with the results: it is not the case that choices of ‘True’ were more likely for ¬∀-$R_{\emptyset}=S$ than ∀-$R_{\emptyset}=S$.

Following the same procedure as for Experiment 1 (see §3.2), I fit a mixed-effect multinomial model with random intercepts for participants and items. Determiner type, nuclear scope type and their interaction were added as fixed effects. Likelihood ratio tests indicated that the addition of each fixed effect produced a significantly better fit, relative to models without that effect (in each case, *p*<0.0001). Within the full model, the estimated coefficients for all levels and their interactions attained significance (see Tables 6 and 7). This indicates that assessors’ choices were affected by determiner type, nuclear scope type and their interaction.

**Table 6:** ‘Can’t say’ vs. ‘False’ in the model for Experiment 2

	*β*	*SE*	*z value*	*p value*
(Intercept)	1.2321	0.4086	3.016	
¬∀	−1.5797	0.4035	−3.915	<0.0001
$R_{\emptyset}$	−0.9875	0.4026	−2.453	0.01418
¬∀:$R_{\emptyset}$	2.8120	0.5244	5.362	<0.0001

**Table 7:** ‘True’ vs. ‘False’ in the model for Experiment 2

	*β*	*SE*	*z value*	*p value*
(Intercept)	2.2285	0.3332	6.687	
¬∀	−5.1281	0.5480	−9.357	<0.0001
$R_{\emptyset}$	−5.5487	0.6854	−8.095	<0.0001
¬∀:$R_{\emptyset}$	8.6645	0.8679	9.983	<0.0001

The estimated marginal means indicated that choices of ‘True’ were significantly more likely for condition ∀-$R_{\emptyset}=S$ than for ¬∀-$R_{\emptyset}=S$ (*MD* = 0.65, *z* = 9.10, *p*<0.0001), and for ¬∀-$R_{\emptyset}$ than for ∀-$R_{\emptyset}$ (*MD* = 0.17, *z* = 3.24, *p* = 0.0066; see Table 20 in the Appendix). This provides support for, respectively, the second prediction of the standard semantics and the first prediction of the alternative semantics.

### Discussion

First, it is worth noting that a surprising result of Experiment 1—the negligible proportion of choices of ‘True’ for empty-restrictor sentences with ‘every’—is replicated in the current results. A comparison of the estimated marginal means for each choice within condition ∀-$R_{ \emptyset}$ indicates that there was a significantly greater likelihood of choices of ‘False’ than ‘True’ (*MD* = 0.42, *z* = 5.70, *p*<0.0001), and of choices of ‘Can’t say’ than ‘True’ (*MD* = 0.54, *z* = 6.69, *p*<0.0001; see Table 22 in the Appendix).

The results conform with the first prediction of the alternative semantics rather than the first prediction of the standard semantics. Hence there does not appear to be empirical support for a clear intuition that sentences of the form ‘Not every A is B’ are true only if there is some A that is not-B. This undermines the argument that each semantic account violates at least one pervasive intuition about empty-restrictor sentences.

One might question the extent to which the results of Experiment 2 support the predictions of the alternative semantics even for sentences in condition ¬∀-$R_{\emptyset}$. For the proportion of choices of ‘True’ in that condition (18.75%) was only slightly higher than that of ‘False’ (15.10%). One possibility is that the falsity of the scalar implicature associated with items of the form ‘Not every A is B’—namely, that some A is B—increases the likelihood of choices of ‘False’ and ‘Can’t say’ (see §4.1). Moreover, the standard semantics are difficult to reconcile with the presence of any non-negligible proportion of choices of ‘True’ in condition ¬∀-$R_{\emptyset}$ (although see §6.2 for an attempted reconciliation). Semantic falsity, an empty restrictor *and* a false implicature should make judgements of falsity or oddness a virtual certainty.

On the other hand, the results violate the predictions of the alternative semantics with respect to empty-restrictor sentences of the form ‘Every A is A’ and ‘Not every A is A’. Experiment 2 is therefore puzzling: the results for $R_{\emptyset}$ conditions contradict a prediction of the standard contemporary semantics and support a prediction of the alternative semantics, whereas the opposite is the case for the results pertaining to $R_{\emptyset}=S$ conditions. All three of the analyses assumed that the nuclear scope would have no effect on choices. In fact, the estimated marginal means for the results indicate that choices of ‘True’ were significantly more likely for condition ∀-$R_{\emptyset}=S$ than for ∀-$R_{\emptyset}$ (*MD* = 0.66, *z* = 9.28, *p*<0.0001), and for condition ¬∀-$R_{\emptyset}$ than for ¬∀-$R_{\emptyset}=S$ (*MD* = 0.16, *z* = 2.95, *p* = 0.0170). Experiment 3 investigates the source of this effect of nuclear scopes.

## Experiment 3

### Background

A distinction is often drawn between *lawlike* and *contingent* occurrences of sentences with ‘every’ (Horn 1977; de Jong and Verkuyl 1984, pp. 29-30; Lappin and Reinhart 1988, pp. 1026-1027; Moravcsik 1991; Heim and Kratzer 1998, pp. 164-169). Two rough hallmarks of lawlike occurrences are that they do not seem to require empirical assessment at the actual world, and they concern a property that is seen as inherent to, or typically associated with, the individuals in the restrictor’s extension. For example, it is natural to understand (3-a) as lawlike and (3-b) as contingent:

(3)
  - Every raven is black.^12^
  - Every raven is sick.

Accounts of the mechanisms that underlie the distinction tend to hold that lawlike occurrences are evaluated relative to non-actual worlds where the restrictor denotes a non-empty set (for further discussion, see §6.3). Assessors are often able to infer that the non-empty sets denoted by restrictors at the relevant worlds are, or fail to be, subsets of the sets denoted by nuclear scopes. For example, relative to any world where American kings exist, the set of American kings will be a subset of the set of American kings, resulting in the truth of (2-a) and the falsity of (2-c).

A reasonable supposition is that the sentences in condition $R_{\emptyset}=S$ are likely to be understood as lawlike (e.g., ‘Every American king is an American king’), and those in condition $R_{\emptyset}$ are likely to be understood as contingent (e.g., ‘Every American king lives in New York’). If so, then the results of Experiment 2 might have been affected by this distinction. Experiment 3 aims to provide evidence that the lawlike-contingent distinction is relevant to the differences between results for conditions ∀-$R_{\emptyset}$ and ∀-$R_{\emptyset}=S$ in Experiment 2.

### Methods

#### Participants

Forty English speakers were recruited through Amazon Mechanical Turk in the same way as for Experiment 1.

#### Materials

I designed an experiment with target empty-restrictor sentences in two test conditions: sentences that are naturally understood as contingent and include a term that makes reference to the actual world (∀-$R^{@}_{\emptyset}$), and sentences that are more likely to be understood as lawlike (∀-$R^{w}_{\emptyset}\subseteq S^{w}$). Each sentence in condition ∀-$R^{@}_{\emptyset}$ was one of the eight sentences from Experiment 1’s condition ∀-$R_{ \emptyset}$ with the addition of one of the following expressions: ‘in the actual world’, ‘actual’, ‘real-life’ or ‘actually’. Each sentence in condition ∀-$R^{w}_{\emptyset}\subseteq S^{w}$ was one of the eight sentences from condition ∀-$R_{\emptyset}$ with the replacement of its nuclear scope with a predicate that included either one of the terms from the restrictor, or an expression that is closely associated with a term in the restrictor and necessarily denotes a superset of that term’s extension. The first two items for each condition are given in Table 8 (see the Appendix for the full list of items and fillers). The values listed are the ones predicted by the different semantics after some kind of supplementation to handle lawlike occurrences.

**Table 8:** First two items for each condition

*Condition*	*Item*	*Sentence*	*Standard*	*Non-standard*	*Aristotelian*
∀-$R^{@}_{\emptyset}$	1	In the actual world, every American king lives in New York	true	undefined	false
∀-$R^{@}_{\emptyset}$	2	Every actual egg-laying cow yields a lot of milk	true	undefined	false
∀-$R^{w}_{\emptyset} \subseteq S^{w}$	9	Every American king is royal	true	true	true
∀-$R^{w}_{\emptyset} \subseteq S^{w}$	10	Every egg-laying cow is a cow	true	true	true

Forty lists were produced in the same manner as for Experiment 1, where each list consisted of all sixteen of the target items (eight in each condition) presented in a randomised order alongside sixteen filler sentences. All target sentences included the determiner ‘every’, since a contrast with other determiners or negated sentences was irrelevant to the experimental aim. Filler items included the determiner ‘no’, in order to ensure adequate attention to determiners. The absence of multiple target items that differed only with respect to the determiner led to the decision to expose all participants to all target items. The items in condition ∀-$R^{@}_{ \emptyset}$ varied according to which of four types of expression pertaining to the actual world they contained. These expressions were included in order to sharpen the contrast between sentences that were intended to be understood as contingent or as lawlike. The aim of including a variety of such expressions was to ensure adequate attention to lexical items. In order to investigate a broader range of sentences that are likely to be understood as lawlike, the items in condition ∀-$R^{w}_{ \emptyset}\subseteq S^{w}$ included two types of such sentence, neither of which were identical with the sentences in Experiment 2’s condition ∀-$R_{\emptyset}=S$.

#### Procedure

The instructions and presentation of surveys were the same as for Experiment 1.

#### Predictions

Experiment 3 aimed to evaluate whether the lawlike-contingent distinction affects value judgements, by testing the following prediction:

- **Lawlike Effect:** Choices of ‘True’ are more likely for ∀-$R^{w}_{\emptyset}\subseteq S^{w}$ than for ∀-$R^{@}_{\emptyset}$.

I focused on choices of ‘True’ across conditions because all accounts that have been supplemented in light of Experiment 1’s results continue to expect some non-negligible proportion of other choices (see §4.2.4).

### Results

**Fig. 3:**
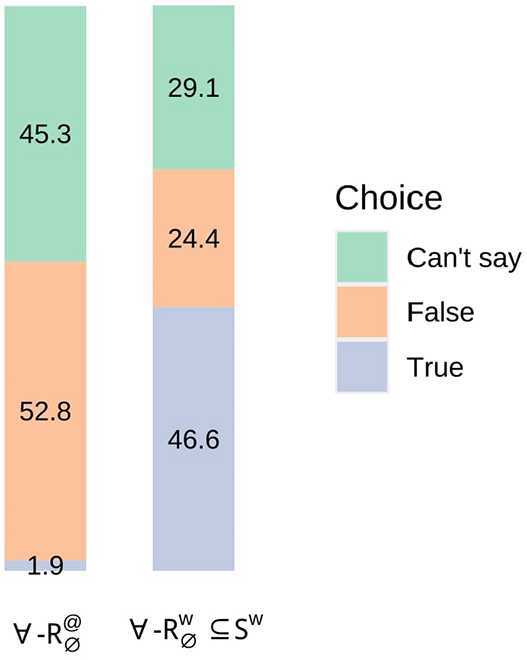
Experiment 3 Results (proportion (%) of choice for each condition; multiplying by 320 and rounding to the nearest integer reconstructs exact counts)

The full results are presented in Fig. 3, and show a higher proportion of choices of ‘True’ for condition ∀-$R^{w}_{ \emptyset}\subseteq S^{w}$ (46.6%) than for ∀-$R^{@}_{ \emptyset}$ (1.9%). The fact that there was a lower proportion of choices of ‘True’ for condition ∀-$R^{w}_{\emptyset}\subseteq S^{w}$ than for condition ∀-$R_{\emptyset}=S$ in Experiment 2 (67.7%) plausibly suggests that more assessors understood more sentences as lawlike in the latter condition—where sentences had the form of tautologies—than in the former.

Following the same procedure as for Experiment 1 (see §3.2), I fit a mixed-effect multinomial model with random intercepts for participants and items, and with condition as a fixed effect. A likelihood ratio test indicated that the addition of the fixed effect produced a significantly better fit (*p*<0.0001). Within the full model, the estimated coefficients for conditions attained significance in the comparison of choices ‘True’ and ‘False’, but not in the comparison of ‘Can’t say’ and ‘False’ (see Tables 9 and 10). Hence condition was a predictor of assessors’ choices, even taking into account differences between participants and items.

**Table 9:** ‘Can’t say’ vs. ‘False’ in the model for Experiment 3

	*β*	*SE*	*z value*	*p value*
(Intercept)	−0.1532	0.2719	−0.563	
∀-$R^{w}_{\emptyset}\subseteq S^{w}$	0.3291	0.2581	1.275	0.202

**Table 10:** ‘True’ vs. ‘False’ in the model for Experiment 3

	*β*	*SE*	*z value*	*p value*
(Intercept)	−3.3381	0.5031	−6.635	
∀-$R^{w}_{\emptyset}\subseteq S^{w}$	3.9854	0.5389	7.396	<0.0001

The estimated marginal means indicate that choices of ‘True’ were significantly more likely for condition ∀-$R^{w}_{\emptyset}\subseteq S^{w}$ than ∀-$R^{@}_{\emptyset}$ (*MD* = 0.45, *z* = 5.93, *p*<0.0001; see Table 21 in the Appendix), supporting a Lawlike Effect.

### Discussion

The negligible proportion of choices of ‘True’ for empty-restrictor sentences with ‘every’ is replicated in the current results, despite the addition of expressions pertaining to the actual world. A comparison of the estimated marginal means for each choice within condition ∀-$R^{@}_{ \emptyset}$ indicates that there was a significantly greater likelihood of choices of ‘False’ than ‘True’ (*MD* = 0.51, *z* = 7.65, *p*<0.0001), and of choices of ‘Can’t say’ than ‘True’ (*MD* = 0.43, *z* = 6.32, *p*<0.0001; see Table 22 in the Appendix).

A reasonable question is whether the differences between conditions ∀-$R^{@}_{\emptyset}$ and ∀-$R^{w}_{\emptyset} \subseteq S^{w}$ could be attributed to the presence of expressions pertaining to the actual world. While this possibility cannot be ruled out, an informal comparison of the results for sentences with and without those expressions suggest that their presence had little impact. For example, the proportion of choices of ‘False’ for condition ∀-$R^{@}_{\emptyset}$ (52.8%) is neither notably higher nor lower than the proportions for condition ∀-$R_{\emptyset}$ in Experiment 1 (66.2%) or in Experiment 2 (43.2%). Of course, a number of difficulties surround the comparison of similar conditions across experiments, such as the potential effects of other sentences that appear in those experiments (see fn. 11). While this means that the results of Experiment 3 should be interpreted with caution, they nevertheless provide some evidence that assessments of empty-restrictor sentences with ‘every’ differ depending on whether those sentences are understood as contingent or lawlike.

Finally, the similar proportion of choices of ‘False’ relative to ‘Can’t say’ for conditions ∀-$R^{@}_{\emptyset}$ and ∀-$R^{w}_{ \emptyset}\subseteq S^{w}$ would be explained by the hypothesis that assessors who made these choices understood the relevant sentences as contingent, and that the likelihood of each participant’s responding to contingent occurrences by assigning the predicted semantic value or experiencing oddness was not significantly affected by nuclear scope type.

## General discussion

The existing literature on empty restrictors motivates three possible accounts of ‘every’: the standard contemporary semantics supplemented with the pragmatic oddness thesis, the non-standard contemporary semantics, and the Aristotelian semantics supplemented with the pragmatic oddness thesis. It appears that none of these accounts can explain the current results without drawing on further resources. The standard and non-standard contemporary accounts do not explain the False ‘Every’ Effect that emerged from Experiment 1. The standard contemporary account does not explain the comparatively high proportion of choices of ‘True’ for sentences of the form ‘Not every A is B’ in Experiment 2. The non-standard contemporary and Aristotelian accounts do not explain the high proportion of choices of ‘True’ for sentences of the form ‘Every A is A’ in Experiment 2; although arguably, this would be addressed by an explanation of the Lawlike Effect. Finally, none of the accounts explain the Lawlike Effect in Experiment 3. I now consider some potential supplements, and evaluate each of the resulting accounts.

### Supplements for Experiment 1

One option for explaining the False ‘Every’ Effect would be to supplement the standard or non-standard contemporary semantics with the following thesis:

- **Pragmatic accommodation:** When 〚NP〛 =∅ and an occurrence of ‘Every NP VP’ presupposes that 〚NP〛 ≠∅, assessors might evaluate an occurrence of ‘Every NP VP’ relative to some context $c'$ where 〚NP〛 ≠∅, and the occurrence might be false at $c'$.

The pragmatic accommodation thesis (suggested by an anonymous reviewer) holds that assessors sometimes evaluate occurrences of empty-restrictor sentences with ‘every’ relative to contexts where the non-emptiness presupposition is satisfied.^13^ For instance, assessors might consider an occurrence of (1-a) relative to a non-actual world where there are American kings, and the occurrence will be false if some of those kings do not live in New York in that world. The potential for this supplement to explain choices of ‘False’ crucially depends on the lexical material used in the items (see §2.2): the same evaluation strategy might be expected to yield choices of ‘True’ for variants of (1-a) with nuclear scopes like ‘lives on Earth’. The thesis fits naturally with the non-standard contemporary semantics, which encode a non-emptiness presupposition as a definedness condition. The thesis is compatible with standard contemporary semantic accounts only if the pragmatic oddness thesis identifies the infelicitous feature as a non-emptiness presupposition separate from the semantics (Reinhart 2004, Geurts 2007).

A second option is to begin by supplementing the standard contemporary semantics with a version of the pragmatic oddness thesis that identifies the infelicitous feature as an implausible conversational implicature. For instance, Abusch and Rooth (2003) hold that (1-a) and (1-b) are asymmetrically entailed by ‘There are no American kings’, so occurrences of (1-a) and (1-b) typically implicate that the speaker does not believe that there are no American kings. The following thesis might then explain the False ‘Every’ Effect:

- **False implicature:** When 〚NP〛 =∅ and an occurrence of ‘Every NP VP’ implicates that the speaker does not believe that 〚NP〛 ≠∅, assessors might judge the occurrence to be false because the implicated speaker belief is false.

That is, assessors who are not trained to disentangle expressed and implicated content might describe a semantically true occurrence of a sentence as ‘false’ on the basis of its implicature (see Spychalska et al. 2016).

A third option is to supplement the non-standard semantics with the following thesis:

- **Pragmatic rejection:** When 〚Every NP VP〛 = undefined and ‘VP’ contains an expression that renders salient an entity (normally a place or individual) that could be examined in order to falsify the occurrence even if 〚NP〛 ≠∅, assessors might ‘pragmatically reject’ the occurrence by judging it to be false.

This thesis (proposed by von Fintel 2004, pp. 292-294) takes the undefinedness of certain occurrences of empty-restrictor sentences with ‘every’ to license a pragmatic strategy for assigning a binary truth value. For instance, ‘New York’ supplies a place that could be examined at the actual world and found to lack American kings, which would falsify the occurrence of (1-a) if the restrictor were actually non-empty. The requirement for undefinedness means that the thesis is incompatible with the standard contemporary semantics.

All three supplements face challenges. First, the results of Experiment 1 pose a challenge for the false implicature thesis. Advocates of the view hold that empty-restrictor sentences with ‘no’ have the same implicature as variants with ‘every’—that the speaker does not believe that the restrictor is empty—and are deemed odd for the same reason.^14^ It seems to follow that assessors who judge occurrences of sentences with ‘every’ to be false due to the implicature would be apt to do the same for variants with ‘no’. This would predict a similar proportion of choices of ‘False’ relative to ‘Can’t say’ for empty-restrictor sentences with ‘every’ and with ‘no’. Yet this prediction is not compatible with the results of Experiment 1 (see §3.3). A fully developed version of the false implicature view would need to predict determiner-based differences.

Next, the results of Experiment 3 appear to violate a prediction of the pragmatic accommodation thesis. Sentences in condition ∀-$R^{@}_{ \emptyset}$ include expressions that favour interpretation relative to the actual world. Hence the pragmatic accommodation thesis might be taken to predict that choices other than ‘False’ are more likely than choices of ‘False’ for condition ∀-$R^{@}_{\emptyset}$. In other words, assessors’ choices should reflect either the semantic values predicted relative to contexts involving the actual world, or oddness responses. Yet the majority of choices for sentences in condition ∀-$R^{@}_{ \emptyset}$ were ‘False’. Granted, Experiment 3 provides no evidence that assessors did consider sentences in condition ∀-$R^{@}_{ \emptyset}$ relative to the actual world. To attain such evidence, future experiments might ask participants to explain their reasoning after they have assessed sentences like those in ∀-$R^{@}_{\emptyset}$ (see §6.4).

The results of Experiment 3 pose another problem for both the pragmatic accommodation and pragmatic rejection theses. It would be natural to attribute choices other than ‘True’ for condition ∀-$R^{w}_{\emptyset} \subseteq S^{w}$ to assessors’ understanding occurrences of sentences as contingent. The problem is that around a quarter of choices for condition ∀-$R^{w}_{\emptyset}\subseteq S^{w}$ were ‘False’. It is difficult to see how pragmatic accommodation could explain this non-negligible proportion: any assessor who accommodates a non-emptiness presupposition for these sentences would surely imagine a world that yielded truth—such as one where all of the American kings *are* royal—rather than falsity. It is also difficult to see how pragmatic rejection could explain this proportion: the nuclear scopes of sentences in condition ∀-$R^{w}_{ \emptyset}\subseteq S^{w}$—‘is royal’, ‘is a cow’, and so on—never include expressions that make an entity salient.^15^ This suggests that advocates of either thesis might be required to endorse further supplements—such as a constrained version of the indistinct choices hypothesis (see §3.3)—in order to explain choices of ‘False’ in condition ∀-$R^{w}_{\emptyset}\subseteq S^{w}$.^16^

### Supplements for Experiment 2

The standard contemporary semantics require further supplementation to explain the non-negligible proportion of choices of ‘True’ for sentences of the form ‘Not every A is B’ in condition ¬∀-$R_{ \emptyset}$. These semantics predict that choices of ‘False’ and ‘Can’t say’ are a near certainty, due to semantic falsity, plus a false implicature (that some A is B), plus an empty restrictor. The only explanation that I am able to think of extends the pragmatic accommodation thesis (see §6.1) to occurrences of empty-restrictor sentences with ‘not every’. On this view, a number of assessors evaluated occurrences relative to contexts where a non-emptiness presupposition is satisfied and truth results, such as a world where there are American kings and some of those kings do not live in New York.

The concern now emerges that a standard contemporary semantic account compatible with the results of Experiments 1 and 2 becomes unamenable to empirical testing, at least via truth value judgement tasks. Such an account holds that occurrences of empty-restrictor sentences with ‘every’ are true, but most assessors will select ‘Can’t say’ (due to the pragmatic feature underlying oddness responses) or ‘False’ (due to pragmatic accommodation or a false implicature); and occurrences of empty-restrictor sentences with ‘not every’ are false, but many assessors will select ‘Can’t say’ (for the same reason as before) or ‘True’ (probably due to pragmatic accommodation). It begins to look as if there is no reliable connection between semantic values and assessors’ evaluations, for any of the sentences that can be used to test the predictions of the standard semantics. This provides motivation to either empirically investigate the prospects for alternative semantic accounts before considering standard contemporary semantic accounts, or to employ a broader range of empirical methods.

### Supplements for Experiment 3

None of the accounts predict the differences between responses to contingent and lawlike occurrences of sentences. These differences emerged most prominently in Experiment 3’s Lawlike Effect, but were also plausibly the source of the failed prediction of the non-standard and Aristotelian semantics in Experiment 2. One option would be to claim that lawlike occurrences involve pragmatic accommodation of a non-emptiness presupposition, leading to evaluation relative to a non-actual world where the restrictor is non-empty:

- **Lawlike pragmatic accommodation:** If an occurrence of ‘Every NP VP’ presupposes that 〚NP〛 ≠∅ and is understood as lawlike, then assessors are likely to evaluate it relative to some world where 〚NP〛 ≠∅.

This approach would be available to theorists independently of whether they accept the pragmatic accommodation thesis for contingent occurrences of sentences.

A second option would take the following form. Based on a suggestion in Diesing (1992), Heim and Kratzer (1998, pp. 168-169) argue that lawlike occurrences of sentences involve implicit modal operators that take scope over quantifier expressions, causing expressions in such sentences to be interpreted relative to non-actual worlds where the restrictor denotes a non-empty set.

- **Lawlike implicit modals:** An occurrence of ‘Every NP VP’ is understood as lawlike if and only if it is understood to involve an implicit modal operator that causes it to be interpreted relative to worlds where 〚NP〛 ≠∅.

Heim and Kratzer do not provide a full technical implementation of this idea, and Reinhart (2004, fn. 31) raises several issues that a developed version would need to address.

There are likely to be important differences between fully elaborated versions of the pragmatic accommodation and implicit modals theses. First, only the former requires that lawlike occurrences trigger non-emptiness presuppositions. Second, only the latter takes the logical form or semantic representation linked to lawlike occurrences to include a component that makes reference to alternate worlds. Third, the implicit modals thesis implies that the truth of lawlike occurrences requires the quantifier relation to hold between the extensions of the restrictor and the nuclear scope at *all* accessible worlds at which the restrictor is non-empty. The pragmatic accommodation thesis implies that an assessor evaluates the occurrence at *some* easily imaginable world where the restrictor is non-empty. Nevertheless, both options predict that assessors will often be able to infer that the non-empty sets denoted by restrictors at the relevant worlds are, or fail to be, subsets of the sets denoted by nuclear scopes. Hence supplementing any semantic account with suitably elaborated versions of either thesis could reconcile them with Experiment 3’s results.

A further idea worth considering is that at least some lawlike occurrences are evaluated via a strategy reserved for tautologous or analytic sentences, where assessors assign values without determining the sub-expressions’ extensions at any world. This strategy might allow assessors to judge ‘Every blue blicket is a blicket’ as true without having any clue about the actual or possible extension of ‘blicket’.^17^ It is unlikely that this idea could explain all lawlike occurrences, given that the paradigm example—‘Every raven is black’—is generally classified as non-analytic (see fn. 12). Still, the possibility remains that multiple evaluation strategies and phenomena underlie the occurrences currently classified under the label ‘lawlike’.

### Evaluating the accounts

Supplements that would allow each of the three semantic analyses of ‘every’ to capture the full results are summarised in Table 11. The standard contemporary semantics additionally require a supplement to explain choices of ‘True’ for the sentences with ‘not every’ in Experiment 2.

**Table 11:** Analyses of ‘Every NP VP’ with empty ‘NP’

*Semantics*	*Oddness responses*	*Descriptions as ‘false’*	*Lawlike occurrences*
*Standard contemporary*	pragmatic oddness	pragmatic accommodation false implicature	pragmatic accommodation implicit modals
*Non-standard contemporary*	-	pragmatic accommodation pragmatic rejection	pragmatic accommodation implicit modals
*Aristotelian*	pragmatic oddness	-	pragmatic accommodation implicit modals

On one hand, standard and non-standard contemporary semantic accounts face the disadvantage of requiring supplements for the False ‘Every’ Effect, where each supplement encounters challenges (see §6.1). Standard contemporary accounts face a further disadvantage by requiring supplementation to handle each of the three sets of experimental results, which has the effect of rendering them unamenable to empirical testing via truth value judgement tasks (see §6.2).

The Aristotelian semantics have the advantage of requiring no supplementation for the False ‘Every’ Effect. Yet their prospects depend on the development of a plausible version of the pragmatic oddness thesis. Perhaps any version of that thesis will face challenges equal to those faced by the supplements for the contemporary semantics.^18^ Theoretical considerations that go beyond the current empirical results raise further challenges for the Aristotelian semantics. These semantics are widely thought to issue incorrect predictions for a range of settings, including when ‘every’ occurs under the scope of quantifiers, under sentential negation, and in intensional contexts. For instance, (4-a) appears to be consistent with (4-c), whereas (4-b) is inconsistent with (4-c); yet the Aristotelian semantics predict that (4-a) and (4-b) are truth-conditionally equivalent:^19^

(4)
  - In every class, the professor responded to every question that was posed from the class.
  - In every class, some questions were posed, and the professor responded to every question that was posed from the class.
  - Though in a number of classes, no questions were posed.

This appears to be a compelling argument against the Aristotelian semantics. However, one puzzling observation is that the same type of argument seems to apply to determiner phrases that are widely acknowledged to entail the non-emptiness of a set, like possessives, partitives and certain strong determiners. For example, variants of (4-a) that replace ‘every question’ with ‘(the) students’ questions’, ‘(all/most/some) of the questions’ and ‘most questions’ seem consistent with (4-c) until the clause ‘some questions were posed’ is added; yet analyses of these determiner phrases typically predict that the variants of (4-a) and (4-b) are truth-conditionally equivalent (see Peters and Westerståhl 2006). Whether this observation motivates the rejection of both the Aristotelian semantics for ‘every’ and the standard semantics for other determiner phrases, or indicates a complication in the argument, is an open question.

On the other hand, an argument may be advanced for upholding the standard contemporary semantics, despite the preceding considerations: ‘logical coherence’ favours them (Peters and Westerståhl 2006). A number of properties differ between the quantifiers that the standard and the alternative semantics take ‘every’ to denote, including definiteness (Barwise and Cooper 1981; Keenan and Stavi 1986), strength (Barwise and Cooper 1981), left downward monotonicity (Ladusaw 1979; van Benthem 1986; Westerståhl 1989), left anti-additivity (Kas 1993) and having certain quantifiers as inner and outer negations (Peters and Westerståhl 2006, p. 25). These might be seen as theoretical costs that are sufficiently grave to outweigh any empirical evidence in favour of the Aristotelian or non-standard contemporary semantics.

One response might emphasise that the semantics assigned to determiners should reflect how they are actually used in natural language. As Westerståhl (2005, p. 8) puts it, a semanticist “is not free to stipulate a meaning for a word just because it is logically simpler than an alternative—the alternative might still be the speakers’ choice, and if this is *clearly* so, the speakers rule”. Another response argues that the logical properties of quantifiers are relevant to natural language semantics only in their capacity to predict properties of lexical items, such as distribution. It might turn out that changes in the relevant logical properties for ‘every’ yield no incorrect predictions about the linguistic properties of ‘every’, so have no significant theoretical costs for linguists.^20^

Further empirical work will be vital in evaluating the different accounts. One useful study would present empty-restrictor sentences alongside follow-up questions that probe participants’ reasoning. The Aristotelian semantics predict that those who describe sentences like (1-a) as ‘false’ will justify this choice by saying something like: ‘There are no American kings’. The pragmatic rejection thesis predicts that those assessors will say: ‘Even if there are American kings, none of them live in New York’. The pragmatic accommodation thesis predicts that they will say: ‘Even if there were American kings, some of them would live outside New York’. Categorising participants’ reports along these lines would help to quantify the proportion of choices of ‘False’ that fit with the predictions of the different accounts. Another experiment might attain evidence that choices of ‘Can’t say’ correlate with oddness responses by pairing target sentences with control items (e.g., ‘There are some American kings’), in order to estimate the proportion of choices of ‘Can’t say’ for empty-restrictor sentences that are attributable to epistemic uncertainty (see §3.1). Another relevant study would seek further data about how untrained assessors label true sentences that have false presuppositions or false implicatures. While some existing experiments investigate this issue (see Jasbi et al. 2019), the interpretation of the results in the current paper would benefit from studies that include the same three response labels. For instance, comparing the proportion of choices of ‘False’ and ‘Can’t say’ for sentences like ‘(Some American cities/Few Martian moons) are on Earth’ might reveal whether the pattern of responses in the current experiments reflect the predictions of the standard semantics supplemented with the false implicature thesis. Finally, experiments that present stimuli alongside contexts would overcome methodological concerns surrounding the presentation of isolated sentences and the lexical differences between conditions (see §3.1). For example, sentences of the form ‘Every (blue/red) circle is in the box’ could be presented alongside visual displays that vary with respect to the presence of circles of each colour, and additionally vary the location of the circles relative to a box. Each item and each context could then serve as part of an item-context pair in empty-restrictor conditions and in non-empty-restrictor conditions.

In sum, the current experimental results do not suffice to adjudicate between the three possible accounts of ‘every’ motivated by the existing literature on empty restrictors. However, the results indicate that all of them require further supplementation. The current section has highlighted some potential supplements and further challenges faced by all of the accounts, along with directions for future research.

## Appendix

### Instructions

Participants in each experiment were presented with the following instructions (with ‘*N*’ replaced by the appropriate number of sentences), adapted from Abrusán and Szendrői (2013): *By answering the following questions, you are participating in an academic study about how speakers judge sentences. Your participation in this research is voluntary. You may decline to answer any or all of the following questions. You may decline further participation, at any time, without adverse consequences. Your anonymity is assured; the researchers who have requested your participation will not receive any personal information about you.* *Your task is to judge N English sentences. If you think a sentence is true, you should click on the ‘TRUE’ button. If you think a sentence is false, you should click on the ‘FALSE’ button. Sometimes, it may happen that you cannot decide. In those cases, you should click on the ‘CAN’T SAY’ button. Please do not dwell on your decision for too long. Here is an example:* *In the above example, it is clear that the sentence is false, so please select ‘FALSE’. Here is another example:* *In this case you would probably feel that you cannot decide whether the sentence is true or false. So please select ‘CAN’T SAY’.* *Please note that some, but not all, of the sentences have a correct answer. We will reject users with error rates higher than 25*% *for the sentences that have a correct answer. The correct answer will be obvious for anyone with basic general knowledge. In order to get paid, please make sure that you answer all N questions.*

### Tables

Table 12 presents the full list of target sentences included in Experiment 1, along with the predictions of the standard semantics. Efforts were made to balance potentially relevant features within conditions, including animacy of subject, the presence of stage-level and individual-level predicates, and the inclusion of expressions that render an entity salient (see Lasersohn 1993; von Fintel 2004). Table 13 presents the target sentences included in Experiment 2, along with the predictions of the standard contemporary semantics and the Aristotelian semantics. Table 14 presents the target sentences included in Experiment 3, along with the predictions issued by the standard and Aristotelian semantics once they have been supplemented to capture the interpretation of lawlike occurrences. Efforts were made to balance the choices of the four expressions pertaining to the actual world across the potentially relevant features described above with respect to Table 12 (animacy, etc.). Tables 15, 16, and 17 present the full list of filler sentences included in (respectively) Experiments 1, 2 and 3, along with the predicted truth values.

**Table 12:** Full list of target items in Experiment 1

*Condition*	*Item*	*Sentence*	*Standard*
∀-$R_{\emptyset}$	1	Every American king lives in New York	true
¬∃-$R_{\emptyset}$	1	No American king lives in New York	true
∀-$R_{\emptyset}$	2	Every egg-laying cow yields a lot of milk	true
¬∃-$R_{\emptyset}$	2	No egg-laying cow yields a lot of milk	true
∀-$R_{\emptyset}$	3	Every six-headed woman has collaborated with Taylor Swift	true
¬∃-$R_{\emptyset}$	3	No six-headed woman has collaborated with Taylor Swift	true
∀-$R_{\emptyset}$	4	Every invisible mountain is in Japan	true
¬∃-$R_{\emptyset}$	4	No invisible mountain is in Japan	true
∀-$R_{\emptyset}$	5	Every blue apple tastes sweet	true
¬∃-$R_{\emptyset}$	5	No blue apple tastes sweet	true
∀-$R_{\emptyset}$	6	Every flying house is owned by Kanye West	true
¬∃-$R_{\emptyset}$	6	No flying house is owned by Kanye West	true
∀-$R_{\emptyset}$	7	Every living unicorn is suffering from diseases	true
¬∃-$R_{\emptyset}$	7	No living unicorn is suffering from diseases	true
∀-$R_{\emptyset}$	8	Every six-thousand-year-old phone is ringing loudly	true
¬∃-$R_{\emptyset}$	8	No six-thousand-year-old phone is ringing loudly	true
∀-*T*	9	Every living person was born on Earth	true
¬∃-*T*	9	No living person was born on Earth	false
∀-*T*	10	Every American president was born male	true
¬∃-*T*	10	No American president was born male	false
∀-*T*	11	Every Apple employee has heard of Steve Jobs	true
¬∃-*T*	11	No Apple employee has heard of Steve Jobs	false
∀-*T*	12	Every Florida orange is grown in the United States	true
¬∃-*T*	12	No Florida orange is grown in the United States	false
∀-*T*	13	Every beef burger includes meat	true
¬∃-*T*	13	No beef burger includes meat	false
∀-*T*	14	Every one-dollar bill features an image of George Washington	true
¬∃-*T*	14	No one-dollar bill features an image of George Washington	false
∀-*T*	15	Every senior citizen is older than a newborn baby	true
¬∃-*T*	15	No senior citizen is older than a newborn baby	false
∀-*T*	16	Every burning tree is on fire	true
¬∃-*T*	16	No burning tree is on fire	false
∀-*F*	17	Every American president was born in China	false
¬∃-*F*	17	No American president was born in China	true
∀-*F*	18	Every black cat is a reptile	false
¬∃-*F*	18	No black cat is a reptile	true
∀-*F*	19	Every famous scientist was fathered by Brad Pitt	false
¬∃-*F*	19	No famous scientist was fathered by Brad Pitt	true
∀-*F*	20	Every Russian spacecraft has crashed in Central Park	false
¬∃-*F*	20	No Russian spacecraft has crashed in Central Park	true
∀-*F*	21	Every international airport is smaller than an ant	false
¬∃-*F*	21	No international airport is smaller than an ant	true
∀-*F*	22	Every ancient pyramid was created by Britney Spears	false
¬∃-*F*	22	No ancient pyramid was created by Britney Spears	true
∀-*F*	23	Every newborn baby is legally able to drive	false
¬∃-*F*	23	No newborn baby is legally able to drive	true
∀-*F*	24	Every maple tree is made of ice	false
¬∃-*F*	24	No maple tree is made of ice	true

**Table 13:** Full list of target items in Experiment 2

*Condition*	*Item*	*Sentence*	*Stand.*	*Arist.*
∀-$R_{\emptyset}$	1	Every American king lives in New York	true	false
¬∀-$R_{\emptyset}$	1	Not every American king lives in New York	false	true
∀-$R_{\emptyset}$	2	Every egg-laying cow yields a lot of milk	true	false
¬∀-$R_{\emptyset}$	2	Not every egg-laying cow yields a lot of milk	false	true
∀-$R_{\emptyset}$	3	Every six-headed woman has collaborated with Taylor Swift	true	false
¬∀-$R_{\emptyset}$	3	Not every six-headed woman has collaborated with Taylor Swift	false	true
∀-$R_{\emptyset}$	4	Every invisible mountain is in Japan	true	false
¬∀-$R_{\emptyset}$	4	Not every invisible mountain is in Japan	false	true
∀-$R_{\emptyset}$	5	Every blue apple tastes sweet	true	false
¬∀-$R_{\emptyset}$	5	Not every blue apple tastes sweet	false	true
∀-$R_{\emptyset}$	6	Every flying house is owned by Kanye West	true	false
¬∀-$R_{\emptyset}$	6	Not every flying house is owned by Kanye West	false	true
∀-$R_{\emptyset}$	7	Every living unicorn is suffering from diseases	true	false
¬∀-$R_{\emptyset}$	7	Not every living unicorn is suffering from diseases	false	true
∀-$R_{\emptyset}$	8	Every six-thousand-year-old phone is ringing loudly	true	false
¬∀-$R_{\emptyset}$	8	Not every six-thousand-year-old phone is ringing loudly	false	true
∀-$R_{\emptyset}=S$	9	Every American king is an American king	true	false
¬∀-$R_{\emptyset}=S$	9	Not every American king is an American king	false	true
∀-$R_{\emptyset}=S$	10	Every egg-laying cow is an egg-laying cow	true	false
¬∀-$R_{\emptyset}=S$	10	Not every egg-laying cow is an egg-laying cow	false	true
∀-$R_{\emptyset}=S$	11	Every six-headed woman is a six-headed woman	true	false
¬∀-$R_{\emptyset}=S$	11	Not every six-headed woman is a six-headed woman	false	true
∀-$R_{\emptyset}=S$	12	Every invisible mountain is an invisible mountain	true	false
¬∀-$R_{\emptyset}=S$	12	Not every invisible mountain is an invisible mountain	false	true
∀-$R_{\emptyset}=S$	13	Every blue apple is a blue apple	true	false
¬∀-$R_{\emptyset}=S$	13	Not every blue apple is a blue apple	false	true
∀-$R_{\emptyset}=S$	14	Every flying house is a flying house	true	false
¬∀-$R_{\emptyset}=S$	14	Not every flying house is a flying house	false	true
∀-$R_{\emptyset}=S$	15	Every living unicorn is a living unicorn	true	false
¬∀-$R_{\emptyset}=S$	15	Not every living unicorn is a living unicorn	false	true
∀-$R_{\emptyset}=S$	16	Every six-thousand-year-old phone is a six-thousand-year-old phone	true	false
¬∀-$R_{\emptyset}=S$	16	Not every six-thousand-year-old phone is a six-thousand-year-old phone	false	true

**Table 14:** Full list of target items in Experiment 3

*Condition*	*Item*	*Sentence*	*Stand.*	*Arist.*
∀-$R^{@}_{\emptyset}$	1	In the actual world, every American king lives in New York	true	false
∀-$R^{@}_{\emptyset}$	2	Every actual egg-laying cow yields a lot of milk	true	false
∀-$R^{@}_{\emptyset}$	3	Every six-headed woman has actually collaborated with Taylor Swift	true	false
∀-$R^{@}_{\emptyset}$	4	Every real-life invisible mountain is in Japan	true	false
∀-$R^{@}_{\emptyset}$	5	In the actual world, every blue apple tastes sweet	true	false
∀-$R^{@}_{\emptyset}$	6	Every real-life flying house is owned by Kanye West	true	false
∀-$R^{@}_{\emptyset}$	7	Every actual living unicorn is suffering from diseases	true	false
∀-$R^{@}_{\emptyset}$	8	Every six-thousand-year-old phone is actually ringing loudly	true	false
∀-$R^{w}_{\emptyset}\subseteq S^{w}$	9	Every American king is royal	true	true
∀-$R^{w}_{\emptyset}\subseteq S^{w}$	10	Every egg-laying cow is a cow	true	true
∀-$R^{w}_{\emptyset}\subseteq S^{w}$	11	Every six-headed woman is female	true	true
∀-$R^{w}_{\emptyset}\subseteq S^{w}$	12	Every invisible mountain can’t be seen	true	true
∀-$R^{w}_{\emptyset}\subseteq S^{w}$	13	Every blue apple is blue	true	true
∀-$R^{w}_{\emptyset}\subseteq S^{w}$	14	Every flying house is a house	true	true
∀-$R^{w}_{\emptyset}\subseteq S^{w}$	15	Every living unicorn has exactly one horn	true	true
∀-$R^{w}_{\emptyset}\subseteq S^{w}$	16	Every six-thousand-year-old phone is six thousand years old	true	true

**Table 15:** Full list of filler items in Experiment 1

*Filler number*	*Sentence*	*Value*
1	Every triangle has three sides	true
2	Every cherry pie contains cherries	true
3	Every oak table is made of wood	true
4	Every European monarch is a cat	false
5	Every Canadian child drinks vodka	false
6	Every galaxy is smaller than a grain of sand	false
7	No green stars are on the American flag	true
8	No ripe banana is made of plastic	true
9	No British child is on the Moon	true
10	No European doctor graduated from college	false
11	No living creatures are in the ocean	false
12	No famous plays were written by Shakespeare	false

**Table 16:** Full list of filler items in Experiment 2

*Filler number*	*Sentence*	*Value*
1	Every cherry pie contains cherries	true
2	Every oak table is made of wood	true
3	Every maple tree is made of ice	false
4	Every Canadian child drinks vodka	false
5	Not every newborn baby will live to the age of eighty	true
6	Not every living creature has legs	true
7	Not every black cat is a mammal	false
8	Not every triangle has three sides	false
9	Every cherry pie is a cherry pie	true
10	Not every oak table is an oak table	false
11	Every maple tree is a maple tree	true
12	Not every Canadian child is a Canadian child	false
13	Every newborn baby is a newborn baby	true
14	Not every living creature is a living creature	false
15	Every black cat is a black cat	true
16	Not every triangle is a triangle	false

**Table 17:** Full list of filler items in Experiment 3

*Filler number*	*Sentence*	*Value*
1	Every cherry pie contains cherries	true
2	Every triangle has three sides	true
3	Every Canadian child drinks vodka	false
4	Every galaxy is smaller than a grain of sand	false
5	No ripe banana is made of plastic	true
6	No British child is on the Moon	true
7	No European doctor graduated from college	false
8	No living creatures are in the ocean	false
9	Every actual oak table is made of wood	true
10	In the actual world, every maple tree is made of ice	false
11	No green stars are actually on the American flag	true
12	No real-life famous plays were written by Shakespeare	false
13	Every maple tree is a tree	true
14	Every ripe banana is ripe	true
15	No Canadian child is a minor	false
16	No European doctor is a doctor	false

Tables 18–21 present all pairwise comparisons between estimated marginal means calculated from the models of Experiments 1–3 in order to evaluate predictions. Table 22 presents the pairwise comparisons contrasting each pair of choices within a key category of empty-restrictor sentences with ‘every’ in each experiment, to evaluate the replication of the negligible proportion of choices of ‘True’.

**Table 18:** Pairwise comparisons of estimated marginal means for Experiment 1, contrasting each pair of conditions for each choice (with Tukey adjustment)

*Contrast*	*Estimate*	*SE*	*z value*	*p value*
*Can’t say*				
¬∃-$R_{\emptyset} - \forall $-$R_{\emptyset}$	−0.0278	0.1176	−0.236	0.9999
¬∃-$R_{\emptyset} - \neg \exists $-*T*	0.2315	0.0939	2.466	0.1342
¬∃-$R_{\emptyset} - \forall $-*T*	0.1806	0.0985	1.833	0.4446
¬∃-$R_{\emptyset} - \neg \exists $-*F*	0.2130	0.0950	2.241	0.2188
¬∃-$R_{\emptyset} - \forall $-*F*	0.2593	0.0931	2.784	0.0600
∀-$R_{\emptyset} - \neg \exists $-*T*	0.2593	0.0842	3.080	0.0253
∀-$R_{\emptyset} - \forall $-*T*	0.2083	0.0917	2.273	0.2051
∀-$R_{\emptyset} - \neg \exists $-*F*	0.2407	0.0872	2.761	0.0639
∀-$R_{\emptyset} - \forall $-*F*	0.2870	0.0835	3.439	0.0077
¬∃-*T* − ∀-*T*	−0.0509	0.0508	−1.002	0.9174
¬∃-*T* − ¬∃-*F*	−0.0185	0.0399	−0.464	0.9973
¬∃-*T* − ∀-*F*	0.0278	0.0256	1.086	0.8873
∀-*T* − ¬∃-*F*	0.0324	0.0549	0.590	0.9917
∀-*T* − ∀-*F*	0.0787	0.0482	1.633	0.5768
¬∃-*F* − ∀-*F*	0.0463	0.0361	1.281	0.7955
*False*				
¬∃-$R_{\emptyset} - \forall $-$R_{\emptyset}$	−0.5000	0.0873	−5.728	<0.0001
¬∃-$R_{\emptyset} - \neg \exists $-*T*	−0.7639	0.0451	−16.938	<0.0001
¬∃-$R_{\emptyset} - \forall $-*T*	0.1204	0.0416	2.890	0.0446
¬∃-$R_{\emptyset} - \neg \exists $-*F*	0.1065	0.0430	2.479	0.1302
¬∃-$R_{\emptyset} - \forall $-*F*	−0.7963	0.0416	−19.133	<0.0001
∀-$R_{\emptyset} - \neg \exists $-*T*	−0.2639	0.0822	−3.212	0.0166
∀-$R_{\emptyset} - \forall $-*T*	0.6204	0.0823	7.538	<0.0001
∀-$R_{\emptyset} - \neg \exists $-*F*	0.6065	0.0832	7.293	<0.0001
∀-$R_{\emptyset} - \forall $-*F*	−0.2963	0.0813	−3.645	0.0036
¬∃-*T* − ∀-*T*	0.8843	0.0299	29.534	<0.0001
¬∃-*T* − ¬∃-*F*	0.8704	0.0322	27.022	<0.0001
¬∃-*T* − ∀-*F*	−0.0324	0.0292	−1.110	0.8773
∀-*T* − ¬∃-*F*	−0.0139	0.0261	−0.533	0.9949
∀-*T* − ∀-*F*	−0.9167	0.0233	−39.422	<0.0001
¬∃-*F* − ∀-*F*	−0.9028	0.0261	−34.532	<0.0001
*True*				
¬∃-$R_{\emptyset} - \forall $-$R_{\emptyset}$	0.5278	0.0992	5.319	<0.0001
¬∃-$R_{\emptyset} - \neg \exists $-*T*	0.5324	0.0994	5.356	<0.0001
¬∃-$R_{\emptyset} - \forall $-*T*	−0.3009	0.1063	−2.832	0.0526
¬∃-$R_{\emptyset} - \neg \exists $-*F*	−0.3194	0.1034	−3.089	0.0246
¬∃-$R_{\emptyset} - \forall $-*F*	0.5370	0.0994	5.401	<0.0001
∀-$R_{\emptyset} - \neg \exists $-*T*	0.0046	0.0181	0.256	0.9999
∀-$R_{\emptyset} - \forall $-*T*	−0.8287	0.0549	−15.105	<0.0001
∀-$R_{\emptyset} - \neg \exists $-*F*	−0.8472	0.0464	−18.268	<0.0001
∀-$R_{\emptyset} - \forall $-*F*	0.0093	0.0172	0.539	0.9946
¬∃-*T* − ∀-*T*	−0.8333	0.0548	−15.204	<0.0001
¬∃-*T* − ¬∃-*F*	−0.8519	0.0462	−18.435	<0.0001
¬∃-*T* − ∀-*F*	0.0046	0.0157	0.294	0.9997
∀-*T* − ¬∃-*F*	−0.0185	0.0660	−0.281	0.9998
∀-*T* − ∀-*F*	0.8380	0.0547	15.327	<0.0001
¬∃-*F* − ∀-*F*	0.8565	0.0460	18.614	<0.0001

**Table 19:** Pairwise comparisons of estimated marginal means for Experiment 1, contrasting each pair of restrictors (averaged over the level of determiner) for each choice (with Tukey adjustment)

*Contrast*	*Estimate*	*SE*	*z value*	*p value*
*Can’t say*				
$R_{\emptyset} - T$	0.2199	0.0663	3.316	0.0026
$R_{\emptyset} - F$	0.2500	0.0654	3.820	0.0004
*T* − *F*	0.0301	0.0304	0.990	0.5833
*False*				
$R_{\emptyset} - T$	−0.0718	0.0468	−1.532	0.2758
$R_{\emptyset} - F$	−0.0949	0.0469	−2.024	0.1065
*T* − *F*	−0.0232	0.0196	1.184	0.4629
*True*				
$R_{\emptyset} - T$	−0.1482	0.0540	−2.744	0.0167
$R_{\emptyset} - F$	−0.1551	0.0526	−2.949	0.0090
*T* − *F*	−0.0069	0.0339	−0.205	0.9772

**Table 20:** Pairwise comparisons of estimated marginal means for Experiment 2, contrasting each pair of conditions for each choice (with Tukey adjustment)

*Contrast*	*Estimate*	*SE*	*z value*	*p value*
*Can’t say*				
∀-$R_{\emptyset}=S - \neg \forall $-$R_{\emptyset}=S$	−0.1510	0.0744	−2.030	0.1770
∀-$R_{\emptyset}=S - \forall $-$R_{\emptyset}$	−0.3021	0.0755	−4.004	0.0004
∀-$R_{\emptyset}=S - \neg \forall $-$R_{\emptyset}$	−0.4115	0.0739	−5.564	<0.0001
¬∀-$R_{\emptyset}=S - \forall $-$R_{\emptyset}$	−0.1510	0.0737	−2.048	0.1704
¬∀-$R_{\emptyset}=S - \neg \forall $-$R_{\emptyset}$	−0.2604	0.0748	−3.483	0.0028
∀-$R_{\emptyset} - \neg \forall $-$R_{\emptyset}$	−0.1094	0.0756	−1.446	0.4705
*False*				
∀-$R_{\emptyset}=S - \neg \forall $-$R_{\emptyset}=S$	−0.4948	0.0722	−6.850	<0.0001
∀-$R_{\emptyset}=S - \forall $-$R_{\emptyset}$	−0.3594	0.0749	−4.797	<0.0001
∀-$R_{\emptyset}=S - \neg \forall $-$R_{\emptyset}$	−0.0781	0.0411	−1.903	0.2269
¬∀-$R_{\emptyset}=S - \forall $-$R_{\emptyset}$	0.1354	0.0720	1.880	0.2364
¬∀-$R_{\emptyset}=S - \neg \forall $-$R_{\emptyset}$	0.4167	0.0654	6.368	<0.0001
∀-$R_{\emptyset} - \neg \forall $-$R_{\emptyset}$	0.2812	0.0676	4.160	0.0002
*True*				
∀-$R_{\emptyset}=S - \neg \forall $-$R_{\emptyset}=S$	0.6458	0.0709	9.104	<0.0001
∀-$R_{\emptyset}=S - \forall $-$R_{\emptyset}$	0.6615	0.0713	9.282	<0.0001
∀-$R_{\emptyset}=S - \neg \forall $-$R_{\emptyset}$	0.4896	0.0704	6.954	<0.0001
¬∀-$R_{\emptyset}=S - \forall $-$R_{\emptyset}$	0.0156	0.0166	0.943	0.7816
¬∀-$R_{\emptyset}=S - \neg \forall $-$R_{\emptyset}$	−0.1562	0.0531	−2.945	0.0170
∀-$R_{\emptyset} - \neg \forall $-$R_{\emptyset}$	−0.1719	0.0531	−3.238	0.0066

**Table 21:** Pairwise comparisons of estimated marginal means for Experiment 3, contrasting the two conditions for each choice (with Tukey adjustment)

*Contrast*	*Estimate*	*SE*	*z value*	*p value*
*Can’t say*				
∀-$R^{@}_{\emptyset} - \forall $-$R^{w}_{\emptyset}\subseteq S^{w}$	0.163	0.0720	2.258	0.0240
*False*				
∀-$R^{@}_{\emptyset} - \forall $-$R^{w}_{\emptyset}\subseteq S^{w}$	0.284	0.0598	4.752	<0.0001
*True*				
∀-$R^{@}_{\emptyset} - \forall $-$R^{w}_{\emptyset}\subseteq S^{w}$	−0.447	0.0754	−5.930	<0.0001

**Table 22:** Pairwise comparisons of estimated marginal means (with Tukey adjustment) contrasting each pair of choices within conditions: ∀-$R_{ \emptyset}$ in Experiment 1, ∀-$R_{\emptyset}$ in Experiment 2, ∀-$R^{@}_{\emptyset}$ in Experiment 3

*Contrast*	*Estimate*	*SE*	*z value*	*p value*
∀*-*$R_{\emptyset}$*, Experiment 1*				
False − Can’t say	0.3519	0.1640	2.145	0.0810
False − True	0.6343	0.0806	7.870	<0.0001
Can’t say − True	0.2824	0.0886	3.187	0.0041
∀*-*$R_{\emptyset}$*, Experiment 2*				
False − Can’t say	−0.1198	0.1506	−0.795	0.7060
False − True	0.4167	0.0730	5.704	<0.0001
Can’t say − True	0.5365	0.0802	6.687	<0.0001
∀*-*$R^{@}_{\emptyset}$*, Experiment 3*				
False − Can’t say	0.0750	0.1326	0.566	0.8383
False − True	0.5094	0.0666	7.648	<0.0001
Can’t say − True	0.4344	0.0688	6.317	<0.0001

## Footnotes

1 Originally from Lappin and Reinhart (1988).

2 Possible exceptions include empty-restrictor sentences with the determiners ‘the’, ‘both’ and ‘neither’, which some (e.g., Barwise and Cooper 1981) have analysed as undefined.

3 A determiner Det is *positive strong* (or *negative strong*, respectively) iff, for any domain *D* and any *A*⊆*D* for which 〚Det〛(*A*) is defined, *A*∈〚Det〛(*A*) (or *A*∉〚Det〛(*A*), respectively); see Barwise and Cooper (1981, p. 182). If Det is not (positive or negative) strong then, Det is *weak*. Some have questioned whether there is a principled basis for classifying determiners in this way (e.g., Keenan and Stavi 1986, pp. 298-299). I remain neutral on this question, and simply use the terms ‘strong’ and ‘weak’ to pick out the two classes of determiners that have been discussed in the literature on empty restrictors. It is sometimes claimed that weak determiners are even less likely to elicit oddness responses when they occur in existential ‘there’-sentences than in ordinary empty-restrictor sentences (e.g., Reinhart 2004), which predicts a difference between assessors’ responses to (1-b) and to ‘There are no American kings living in New York’. In a pilot study, I attempted to detect such an effect by including three groups of empty-restrictor sentences, which varied with respect to whether the first expression was ‘every’, ‘no’, or ‘there are no’. Since the results did not show significant differences between the latter two conditions, I focused on sentences without ‘there’ in the full experiments.

4 As discussed by Lappin and Reinhart (1988, p. 1027), there is good reason to avoid incorporating non-emptiness presuppositions into the semantics of weak determiners. Hence an advocate of this approach must either attribute the oddness of occurrences of sentences with weak determiners to some pragmatic feature, or claim that weak determiners are ambiguous between a weak construal and a strong-like one (see Milsark 1977, Diesing 1992) such that the latter includes a non-emptiness presupposition as a definedness condition.

5 As far as I am aware, the only recent theorist to have advocated the Aristotelian semantics is Moravcsik (1991). He also claims that ‘lawlike’ occurrences of empty-restrictor sentences with ‘every’ receive the value true, a position that attains empirical support below (see §5). Knowlton (2021) develops an account of the mental representation of ‘every’ that entails the non-emptiness of the restrictor; although this is a side-effect of the use of a second-order variable that ranges over ‘groups’, where ‘groups’ are assumed to include one or more things. Knowlton’s analysis is otherwise quite different from both the Aristotelian and the contemporary semantics.

6 See Tian and Breheny (2015, p. 28). Also see discussion of the reduced N400 effects in event-related brain potentials for open-class words that occur later in isolated sentences (e.g., Kutas et al. 2006, p. 667).

7 Payment was contingent upon answering all questions and attaining at least 75% accuracy with respect to ‘catch’ filler items (e.g., ‘No green stars are on the American flag’). This was to ensure sufficient concentration and effort.

8 This point is important in ruling out a potential response to the lack of evidence for a Strength Contrast Effect. This response would claim that it is the total proportion of choices of ‘Can’t say’ and ‘False’ that reflects the proportion of oddness responses (as per the indistinct choices hypothesis). The significant difference between these proportions for conditions ∀-$R_{\emptyset}$ and ¬∃-$R_{\emptyset}$ would support the contrast discussed in the literature, whereby more assessors assign the predicted value for empty-restrictor sentences with ‘no’ than for ones with ‘every’ (either because the pragmatic feature that yields oddness responses is absent for some occurrences of empty-restrictor sentences with ‘no’, or because ‘no’ has a weak (along with a strong-like) disambiguation; see fn. 4). This response is undercut by the preceding considerations.

9 To see the tension between these intuitions, suppose ‘A’ is an empty restrictor and ‘Every A is B’ is false. Then either ‘Not every A is B’ is true even though there is no A that fails to be B, or ‘Not every A is B’ is false relative to a context where ‘Every A is B’ is also false.

10 I am grateful to an anonymous reviewer for emphasising this point. Another potential complication is that Bott et al. (2019) provide evidence of processing difficulties and errors when what they call ‘empty-set quantifiers’ combine with empty nuclear scopes. While they do not directly test ‘not every’, their theory predicts that it belongs to this class. Hence we might expect the same difficulties to affect the evaluation of sentences like (2-c) and the results for condition ¬∀-$R_{ \emptyset}=S$. To quantify any such effect, future experiments might compare sentences in condition ¬∀-$R_{\emptyset}=S$ with variants that include non-empty-set quantifiers like ‘more than five’.

11 The fact that more choices for sentences in condition ∀-$R_{\emptyset}$ were ‘Can’t say’ than ‘False’, whereas the reverse was the case when the same sentences were presented in Experiment 1, could plausibly be attributed to the other sentences that appeared in the two experiments. The processing of sentences with ‘not every’ is likely to be more taxing than the processing of ones with ‘no’. For, first, such sentences involve negation operators, which are known to be fairly taxing (see Wales and Grieve 1969; Arroyo 1982; Tian and Breheny 2015). Second, they are associated with false scalar implicatures, which may have affected participants’ evaluation strategies (see below). It is possible that encountering large numbers of taxing sentences in a single session induces fatigue or confusion, which might increase the likelihood of choices of ‘Can’t say’ for items in certain conditions.

12 Originally from de Jong and Verkuyl (1984). (3-a) illustrates that a lawlike occurrence need not be a candidate for an analytic sentence. Hence criticisms of the analytic-synthetic distinction (e.g., Quine 1951) are not relevant.

13 A similar idea, suggested by a different anonymous reviewer, is that assessors may evaluate the dynamic conjunction of the non-emptiness presupposition and the literal content, and conclude that falsity results. As far as I can tell, the two ideas would predict the same responses in each case. The choice between them may just depend on whether one prefers a static or dynamic analysis of accommodation.

14 Abusch and Rooth (2003) attribute choices of ‘True’ for empty-restrictor sentences with ‘no’ to hearers’ charitably interpreting the speaker to be attending to her salient beliefs about a restricted scenario (say, New York and its inhabitants). They argue that this interpretation strategy is available only for sentences with weak determiners, which explains why such sentences are (supposedly) more likely to be assigned values than ones with strong determiners.

15 When presenting a version of the pragmatic rejection strategy, von Fintel (2004, p. 286) states that a sentence like ‘Every king of France is bald’ ‘does not make salient or even mention the set of bald people in the world’. He contrasts this with sentences like ‘Every king of France is among the bald people in this world’.

16 An anonymous reviewer suggests an interesting predictive model that aims to capture the results of Experiment 1, based on an assumed distinction between strong Logical Forms (‘LFs’) that encode non-emptiness presuppositions and weak LFs that do not: (i) for sentences of the form ‘Every NP VP’, select a strong LF; (ii) for sentences of the form ‘No NP VP’, select a strong LF with probability *p* and a weak LF with probability 1 − *p*; (iii) given a strong LF for ‘Det NP VP’ that attains the value undefined, select the label ‘Can’t say’ with probability *q* and the label ‘False’ with probability 1 − *q*; (iv) given a weak LF for ‘Det NP VP’, select the label ‘True’ if the value is true, ‘False’ if false, and ‘Can’t say’ if undefined. Clauses (i) and (ii) would follow if strong determiners always encode non-emptiness presuppositions, and weak determiners have a strong-like disambiguation that encodes such presuppositions along with a weak disambiguation that does not (see fn. 4). This predictive model could account for the different proportions of choices of ‘True’ for conditions ∀-$R_{ \emptyset}$ and ¬∃-$R_{\emptyset}$: the former condition is predicted to always involve strong LFs (by clause (i)) which will yield choices of ‘Can’t say’ and ‘False’ (by (iii)), while the latter condition is predicted to involve some strong LFs and some weak LFs (by (ii)) such that the weak LFs will always yield choices of ‘True’ (by (iv), due to the truth-conditions predicted by Generalized Quantifier Theory). However, this predictive model could not account for the different relative proportions in the combined total of choices of ‘Can’t say’ and ‘False’ for conditions ∀-$R_{\emptyset}$ and ¬∃-$R_{\emptyset}$, as revealed in the model’s coefficients when countering the indistinct choices hypothesis (see §3.3): for both conditions, these choices are predicted to emerge from strong LFs, and the probability that these LFs will yield choices of ‘Can’t say’ versus ‘False’ is insensitive to determiners (by (iii)). One response might be to formulate different versions of clause (iii) for ‘every’ and ‘no’, perhaps by adapting one of the supplements for the non-standard contemporary semantics mentioned in Sect. 6.1.

17 I thank an anonymous reviewer for suggesting this evaluation strategy and example.

18 One version of the pragmatic oddness thesis—the version that I consider most plausible—would produce an account that is difficult to distinguish empirically from the non-standard contemporary semantics with the pragmatic rejection supplement. This version holds that a presupposition that the restrictor is non-empty arises if and only if the restrictor or determiner phrase is understood to be topical, with the failure of this presupposition’s yielding a sense of oddness (see Ebert and Ebert 2013 for a similar view). An assessor will find (1-a) odd if she understands ‘every American king’ or ‘American king’ to be topical (and construes the sentence as contingent), otherwise assigning the predicted truth value. Like an account supplemented with pragmatic rejection, the resulting Aristotelian account predicts that the likelihood that an assessor will judge an empty-restrictor sentence to be false rather than odd is increased by the inclusion of an expression that makes an entity salient, since such an expression is often a preferred topic.

19 I am grateful to an anonymous reviewer for providing this example.

20 For instance, strength has primarily been used to predict the acceptability of determiner phrases in the post-verbal position of existential ‘there’-sentences (Barwise and Cooper 1981), yet doubts have been raised about the success of such a use (e.g., Keenan 2003). Hence classifying the quantifier denoted by ‘every’ as weak rather than positive strong might entail no incorrect predictions about the distribution of ‘every’.
